# Allelic Variation at the Vernalization Response (*Vrn-1*) and Photoperiod Sensitivity (*Ppd-1*) Genes and Their Association With the Development of Durum Wheat Landraces and Modern Cultivars

**DOI:** 10.3389/fpls.2020.00838

**Published:** 2020-06-23

**Authors:** Conxita Royo, Susanne Dreisigacker, Jose Miguel Soriano, Marta S. Lopes, Karim Ammar, Dolors Villegas

**Affiliations:** ^1^Sustainable Field Crops Programme, Institute for Food and Agricultural Research and Technology (IRTA), Lleida, Spain; ^2^International Maize and Wheat Improvement Center (CIMMYT), Texcoco, Mexico

**Keywords:** heading, anthesis, flowering time, *Vrn-A1*, *Vrn-B1*, *Ppd-A1*, *Ppd-B1*, *Deletion Capelle-Desprez* (*DelCD*)

## Abstract

Wheat adaptability to a wide range of environmental conditions is mostly determined by allelic diversity within genes controlling vernalization requirement (*Vrn-1*) and photoperiod sensitivity (*Ppd-1*). We characterized a panel of 151 durum wheat Mediterranean landraces and 20 representative locally adapted modern cultivars for their allelic composition at *Vrn-1* and *Ppd-1* gene using diagnostic molecular markers and studied their association with the time needed to reach six growth stages under field conditions over 6 years. Compared with the more diverse and representative landrace collection, the set of modern cultivars were characterized by a reduction of 50% in the number of allelic variants at the *Vrn-A1* and *Vrn-B1* genes, and the high frequency of mutant alleles conferring photoperiod insensitivity at *Ppd-A1*, which resulted on a shorter cycle length. *Vrn-A1* played a greater role than *Vrn-B1* in regulating crop development (*Vrn-A1* > *Vrn-B1*). The results suggest that mutations in the *Vrn-A1* gene may have been the most important in establishing the spring growth habit of Mediterranean landraces and modern durum cultivars. The allele *Vrn-A1d*, found in 10 landraces, delayed development. The relative effects of single *Vrn-A1* alleles on delaying the development of the landraces were *vrn-A1* = *Vrn-A1d* > *Vrn-A1b* > *Vrn-A1c*. Allele *vrn-B1* was present in all except two landraces and in all modern cultivars. The null allele at *Ppd-A1* (a deletion first observed in the French bread wheat cultivar ‘Capelle-Desprez’) was found for the first time in durum wheat in the present study that identified it in 30 landraces from 13 Mediterranean countries. Allele *Ppd-A1a* (*GS105*) was detected in both germplasm types, while the allele *Ppd-A1a* (*GS100*) was found only in modern North American and Spanish cultivars. The relative effect of single *Ppd-A1* alleles on extending phenological development was *Ppd-A1*(*DelCD*) > *Ppd-A1b* > *Ppd-A1a* (*GS105*) > *Ppd-A1a* (*GS100*). Sixteen *Vrn-1+Ppd-1* allelic combinations were found in landraces and six in modern cultivars, but only three were common to both panels. Differences in the number of days to reach anthesis were 10 days in landraces and 3 days in modern cultivars. Interactive effects between *Vrn-1* and *Ppd-1* genes were detected.

## Introduction

Flowering time is a major adaptive trait of wheat that allows the crop to fill the grains under the most favorable environmental conditions, so it has a strong effect on reproductive success and final yield (reviewed by Cockram et al., 2007). In environments such as the Mediterranean, where temperatures rise and water becomes scarce during grain filling, early flowering is a very efficient mechanism for escaping terminal stress.

It is widely accepted that during domestication of wheat about 10,000 years before the present and the spread of domesticated wheat, adaptive traits suited to the new environments were selected (Charmet, 2011; Peng et al., 2011). Fitting flowering time to the prevailing environmental conditions in a given region was probably one of the most critical features that facilitated the colonization of new environments during this dispersal (Cockram et al., 2009). Mediterranean wheat landraces resulted from the migration from the Fertile Crescent to the west and were grown in the region for thousands of years until the advent of the more productive semi-dwarf cultivars arising from the Green Revolution in the early 1970s.

The complex process of wheat development is determined to a great extent by the allelic diversity within genes regulating the vernalization requirement (*Vrn*) and photoperiod sensitivity (*Ppd*). A third group of loci controlling ‘narrow-sense earliness’ or ‘earliness *per se*’ (*Eps*) is characterized by a polygenic inheritance and acts on the developmental rate independently of vernalization and photoperiod constituents (van Beem et al., 2005). The molecular basis of the complex genetic regulation of flowering time in wheat has been extensively studied, but there is still considerable uncertainty about the phenotypic response of the plants due to the interactive effect of the genes involved.

Vernalization is the induction of the flowering process by exposure to cold. According to their need for vernalization in order to become capable of flowering, wheat plants can be categorized as winter types (a strong vernalization requirement), spring types (no vernalization requirement) or facultative types (an intermediate growth habit). The vernalization requirement of durum wheat (*Triticum turgidum* L. ssp. *durum*), a tetraploid species with the AABB genome, is mainly determined by allelic variation at two *Vrn* genes: *Vrn-1* (Yan et al., 2003) and potentially *Vrn-3* (Yan et al., 2006). Durum wheat contains homologous copies of *Vrn-1*, named *Vrn-A1* and *Vrn-B1* and positioned on the middle of the long arms of chromosomes 5A and 5B, respectively (Yan et al., 2004; Fu et al., 2005). *Vrn-1* encodes a MADS-box transcription factor, which is involved in the transition of the apical meristem from the vegetative to the reproductive stages (Danyluk et al., 2003; Trevaskis et al., 2003; Yan et al., 2003).

Several alleles have been described within *Vrn-A1* and *Vrn-B1* on the basis of their special structural characterizations and vernalization requirements, as well as their effect on flowering time. A dominant allele for any of the *Vrn* genes leads to a spring growth habit regardless of the allelic state of the other genes, but the presence of recessive alleles for the two *Vrn-1* genes leads to a winter growth habit (Turner et al., 2013). In tetraploid and hexaploid wheat the dominant *Vrn-A1* allele for spring growth-habit carries either mutations in the promoter region or a large deletion within the first intron of a recessive *vrn-A1* allele for the wild-type winter growth-habit (Yan et al., 2004; Fu et al., 2005; Dubcovsky et al., 2006; Pidal et al., 2009). The promoter region of the *Vrn-A1a* allele is duplicated, while the *Vrn-A1b* allele differs from the *vrn-A1* allele in a 20-bp deletion in the TC-repetitive elements of the 50 untranslated region. The alleles *Vrn-A1c* (a 7,222-bp deletion in the intron1 region), *Vrn-A1d* (a 32-bp deletion in the promoter region) and *Vrn-A1e* (a 54-bp deletion in the promoter region) have been described in tetraploid wheat (Yan et al., 2004; Fu et al., 2005). The dominant *Vrn-B1* alleles are mainly caused by the large deletions within the first intron (Fu et al., 2005; Milec et al., 2013; Muterko et al., 2016a). The dominant *Vrn-A1a* allele has the most marked genetic effect on the development of a spring habit, as plants bearing this allele require no cold treatment at all to flower (Stelmakh, 1998; Zhang et al., 2008). The *Vrn-B1* gene has a much lower effect than *Vrn-A1* on vernalization requirement. The presence of *Vrn-B1* alleles alone has been associated with some residual vernalization response and later flowering (facultative type) (Stelmakh, 1998). Natural variation for *Vrn-3* has been found only in the B genome (Yan et al., 2006; Chen et al., 2013). The *Vrn-B3* gene is the wheat ortholog of the *Arabidopsis* gene *FLOWERING LOCUS T*).

Provided that any requirement for vernalization is met, photoperiod-sensitive wheat can only flower after been exposed to long-days. There are two known genes of photoperiod response in durum wheat, *Ppd-A1* and *Ppd-B1*, located on chromosomes 2AS and 2BS, respectively (Laurie, 1997). Photoperiod insensitivity in durum wheat is caused by mutations in those genes. An ‘*a*’ suffix (e.g., *Ppd-A1a*, McIntosh et al., 2003) designates alleles conferring photoperiod insensitivity, while a ‘*b*’ suffix is by convention assigned to wild-type alleles. Wilhelm et al. (2009) identified two large deletions within the *Ppd-A1* gene in durum wheat (1,027 and 1,117 bp), designated as alleles ‘*GS100*’ and ‘*GS105’*, respectively. They remove a common region from the wild-type sequence and cause photoperiod insensitivity, thus accelerating flowering (Wilhelm et al., 2009). The high frequency of these mutations in modern cultivars and its absence in wild tetraploid wheat led to the conclusion that they arose during wheat domestication (Bentley et al., 2011). The *Ppd-B1* locus was first mapped in bread wheat (Hanocq et al., 2004; Mohler et al., 2004) and later confirmed in durum wheat (Maccaferri et al., 2008). In tetraploid wheat the allele conferring photoperiod sensitivity at *Ppd-A1* has a stronger effect than that at *Ppd-B1* (*Ppd-A1b* > *Ppd-B1b*), and the effect of photoperiod insensitive alleles has been classified as *Ppd-A1a* (*GS100*) > *Ppd-A1a* (*GS105*) > *Ppd-B1a* (Royo et al., 2016).

A series of molecular markers have been developed, mostly for bread wheat, to identify different vernalization response (Yan et al., 2003, 2004; Fu et al., 2005) and photoperiod sensitivity alleles (Kiss et al., 2014 and references herein). Models for the genetic control of flowering in cereals suggest a functional relationship between the vernalization- and photoperiod-response genes *Vrn-1* and *Ppd-1* (Distelfeld et al., 2009). Although a large number of wheat studies report the genetic composition and effects of individual alleles at the *Vrn-1* and *Ppd-1* loci, data on the interactive effects of allele combinations *Vrn-1* + *Ppd-1* on crop development are scarce even for bread wheat (Blake et al., 2009; Iqbal et al., 2011; Diaz et al., 2012), and results of experiments conducted under field conditions are often contradictory (Snape et al., 1985; Worland, 1996; Worland et al., 1998; Kato et al., 2000).

This study was carried out on a representative collection of Mediterranean landraces and a small set of modern durum wheat cultivars adapted to the Spanish environments with the aim of: (*i*) characterizing the major alleles of the *Vrn-1* and *Ppd-1* genes using molecular markers; (*ii*) investigating their geographical distribution; and (*iii*) analyzing possible relationships between the alleles and their combinations in the pattern of development under field conditions. As far as we know, this is the first study examining the allele frequency and distribution of genes associated with responses to vernalization requirement and photoperiod sensitivity in durum wheat landraces and modern cultivars and their effect on phenological development under field conditions.

## Materials and Methods

### Plant Material

We tested a panel of 171 durum wheat lines, including 151 landraces from 21 Mediterranean countries and 20 locally adapted modern cultivars of diverse origins (Supplementary Table S1). The objective was to sample a large portion of the unexplored genetic diversity of ancient durums from the Mediterranean Basin (Soriano et al., 2016) maximizing the variability and representativeness of the region. The small set of locally adapted modern cultivars was included for comparison, as a group of lines representative of the main germplasm pools currently cultivated in the low latitude regions of the world: Mediterranean, CIMMYT (International Maize and Wheat Improvement Center)-derived and South-western US cultivars (Royo et al., 2009). The landraces were selected based on their genetic variability from a larger collection coming from public gene banks (Centro de Recursos Fitogenéticos INIA-Spain, ICARDA Germplasm Bank and USDA Germplasm Bank) complying with the Convention on the Trade in Endangered Species of Wild Fauna and Flora^1^. The landraces were bulk-purified to select the dominant type (usually with a frequency above 80% of the bulk) and the seed was increased in plots planted in the same field in the years before each experiment to ensure a common origin for seeds of all lines.

### Experimental Field Setup

Field experiments were carried out in the 2007, 2008, 2009, 2013, 2014, and 2015 harvesting seasons in Lleida (41°40′N, 0°20′E, 260 m.a.s.l.), northeastern Spain. The experiments were carried out in a non-replicated modified augmented design with three replicated checks (*cv* ‘Claudio,’ ‘Simeto,’ and ‘Vitron’) the first 3 years, and two replicated checks (*cv* ‘Avispa’ and ‘Euroduro’) for the last three, at a ratio of 1:5 between checks and tested genotypes. The plots measured 6 m^2^ in the first 3 years and 3.6 m^2^ in the last three. In all experiments, the plots comprised eight rows spaced 0.15 m apart. Sowing density was adjusted to 250 germinable seeds m^–2^. Planting dates were 21-November 2006, 20-November 2007, 20-November 2008, 4-December 2012, 27-November 2013, and 21-November 2014. Average minimum and maximum monthly temperatures and water rainfall were calculated from daily data recorded for a weather station close to the experimental fields (Supplementary Figure S1). Field management practices during the experiments were in accordance with the standard agronomic practices commonly used in the area.

### Monitoring of Phenological Development

In all the experiments, the plants of each plot were monitored on a twice-weekly basis to record the following growth stages (Zadoks et al., 1974): GS55 (heading), GS65 (anthesis), and GS87 (physiological maturity). Additionally, in the experiments conducted from 2007 to 2009, GS31 (beginning of jointing), GS33 (mid-jointing), and GS45 (booting) were also determined. A plot was considered to have reached a given developmental stage when approximately 50% of the plants exhibited the stage-specific phenotypic characteristics.

### DNA Extraction and Diagnostic Markers for *Vrn-1* and *Ppd-1*

The collection was characterized with a set of molecular markers associated with all *Vrn-1*, *Vrn-3*, and *Ppd-1* genes reported at the time when the analyses were carried out (Supplementary Table S2). Leaf tissue from 5 to 10 plants per entry was collected and DNA isolated using a modified cetyltrimethyl-ammonium bromide (CTAB) procedure, as described in Dreisigacker et al. (2016). Initially we used sequence-tagged-sites (STS), simple sequence repeats (SSR), and single-nucleotide polymorphism (SNP) markers associated with identified polymorphisms in durum wheat, and additional markers for known bread wheat alleles were tested afterward (Supplementary Table S3). *Vrn-1* and *Vrn-3* genetic loci (*Vrn-A1*, *Vrn-B1*, and *Vrn-B3*) were characterized to determine the spring or winter growth habit of each genotype. The gene-specific STS markers described by Yan et al. (2004) and Fu et al. (2005) were used to identify dominant spring alleles due to variation in the promoter and intron-1 region of the *Vrn-A1* gene. In addition, the presence of a SNP, identified so far only in bread wheat (Eagles et al., 2011), and located in Exon 4 of *Vrn-A1* was tested. The methodology described in Fu et al. (2005); Yan et al. (2006), and Chu et al. (2011) was used to identify deletion alleles affecting the vernalization response in the intron-1 region of *Vrn-B1* and *Vrn-B3*. The 1027 bp ‘*GS100*’ type and 1117 bp ‘*GS105*’ type deletions at *Ppd-A1* in durum wheat were detected by applying two Kompetitive Allele Specific PCR (KASP) assays (Wilhelm et al., 2009). The presence of the bread wheat 1.2 kb insertion and the 306 bp deletion at *Ppd-A1* were tested using as controls *cv* ‘Chinese Spring’ and *cv* ‘Cappelle-Desprez,’ respectively (Beales et al., 2007). Linked SSR markers *gwm148* and *gwm257* were initially utilized as described in Hanocq et al. (2004) for *Ppd-B1*. Gene-specific SNPs determining truncated copies, transposon-junction and allele-specific SNPs observed in bread wheat *cv* ‘Sonora64’ (containing three copies of *Ppd-B1*), *cv* ‘Chinese Spring’ (carrying four copies of *Ppd-B1*) and *cv* ‘Cheyenne’ (carrying one copy of *Ppd-B1*) were tested to determine whether similar allele variation exists in durum wheat (Diaz et al., 2012). Copy number variation of *Ppd-B1* alleles could not be identified at the time when the genotypic analyses were carried out. Quantitative techniques for identifying the possible number of copies were not standardized at that time. The photoperiod-insensitive allele was designated *Ppd-1a*, while the alternative allele, which was assumed to infer some photoperiod sensitivity, was designated *Ppd-1b* (Beales et al., 2007).

### Marker Amplification and Visualization

Polymerase chain reaction (PCR) amplifications were performed in a total volume of 10 μl containing a final concentration of 1× Buffer with Green Dye (Promega Corp., United States), 200 μM deoxynucleotide triphosphates (dNTPs), 1.2 mM magnesium chloride (MgCl_2_), 0.25 μM of each primer, 1U of DNA polymerase (GoTaq^®^Flexi, Promega Corp., Cat. # M8295) and 50 ng of DNA template. PCR conditions were performed using the following temperature profile: 94°C for 2 min followed by 30 cycles of 94°C for 1 min, 54–60°C for 2 min (dependent on the primer) and 72°C for 2 min. PCR amplified products were separated by electrophoresis on 1.2% agarose gels using 1× TAE buffer, visualized under UV light. The SNP polymorphisms were scored using KASP reagents^2^. Reactions contained 2.5 mL of water, 2.5 mL of 2× KASPar reaction mix, 0.07 mL of assay mix and 50 ng of dried DNA with a PCR profile of 94°C for 15 min activation time, followed by 20 cycles of 94°C for 10 s, 57°C for 5 s, and 72°C for 10 s, followed by 18 cycles of 94°C for 10 s, 57°C for 20 s and 72°C for 40 s. Fluorescence was read as an end point reading at 25°C.

### Statistical Analysis

Phenological data were fitted to a linear mixed model and restricted maximum likelihood (REML) was used to estimate the variance components and to produce the best linear unbiased predictors (BLUPs). Data were analyzed with the MIXED procedure of the SAS statistical package (SAS Institute Inc., Cary, NC, United States), with the Kenward–Roger correction because of the unbalanced number of cultivars for each allele variant and their combinations. The significance of the differences between the landraces and modern cultivars was assessed by partitioning the sum of squares of the genotype effect into differences between the two types of germplasm and differences within them, which was used as error term. Further analyses were performed sequentially and separately for landraces and modern cultivars considering the year and the gene or gene combinations as fixed factors. In order to assess differences between alleles within each gene, the first analyses were conducted independently for the *Vrn-A1*, *Vrn-B1*, *Ppd-A1*, and *Ppd-B1* genes. In a second step, we studied the combinations of allele variants across the *Vrn* and *Ppd* genes. In the third analyses, combinations of *Vrn* and *Ppd* genes together were examined. The SS of the genotype effect in the ANOVA was partitioned in each case into differences between genes or gene combinations and the genotypic variance retained within them, which was used as error term. Means were compared using the Tukey test at *P* < 0.05.

## Results

### Phenotypic Variation

The number of days from sowing to each growth stage was higher in the landraces than in the modern cultivars, with differences being statistically significant from mid-jointing (Table 1). In relative terms, differences between the two groups in the time needed to reach a given growth stage were 1.8, 3.6, 4.6, 3.4, and 1.2% for GS33, GS45, GS55, GS65, and GS87, respectively.

**TABLE 1:** Average number of days ± SE from sowing to GS31 (beginning of jointing), GS33 (mid-jointing), GS45 (booting), GS55 (heading), GS65 (anthesis), and GS87 (physiological maturity) for the 151 durum wheat landraces and 20 modern cultivars analyzed in this study.

	GS31	GS33	GS45	GS55	GS65	G87
Landraces	124.9 ± 0.15^a^	140.3 ± 0.21^a^	150.3 ± 0.19^a^	153.7 ± 0.22^a^	159.3 ± 0.23^a^	191.3 ± 0.32^a^
Modern	124.2 ± 0.29^a^	137.8 ± 0.33^b^	144.9 ± 0.24^b^	146.6 ± 0.50^b^	153.8 ± 0.58^b^	188.9 ± 0.92^b^

*Data from GS31 to GS45 are means across 3 years and those from GS55 to GS87 are means across 6 years. Means within columns with the same letters are not significantly different for a Tukey test at *P* < 0.05.*

### Distribution Frequency of the *Vrn-1* and *Ppd-1* Alleles in the Collection

Of the 23 allele variants of vernalization and photoperiod genes observed in durum wheat (Supplementary Table S2), 11 were found in landraces and eight in modern cultivars (Table 2). Of the 14 molecular markers applied, seven markers were monomorph, including the marker associated to *Vrn-B3*. Six allele variants of *Vrn-1* were identified in the landraces and only three in the modern cultivars. The winter allele *vrn-A1* was found to be present in only two landraces, but *vrn-B1* was identified in 99% of the landraces and in all modern cultivars. Allele *Vrn-A1c* was by far the most frequent in the modern cultivar group. In the landraces group, *Vrn-A1b* and *Vrn-A1c* were similarly represented, together present in 92% of the landraces (Table 2). We identified five allele variants of *Ppd-1* in both germplasm groups. The null allele (*DelCD* at *Ppd-A1*), a deletion first observed in the bread wheat *cv* ‘Capelle-Desprez,’ was observed for the first time in durum wheat in this study. It was found in 20% of the landraces, but not in any of the modern cultivars used herein. Only a very small fraction (1.3%) of the landraces exhibited a photoperiod insensitive allele at *Ppd-A1*, with the *Ppd-A1a* (*GS100*) allele being completely absent. In the group of modern cultivars, both sensitive and insensitive alleles were present (Table 2). Alleles at *Ppd-B1* were similarly distributed in both groups.

**TABLE 2:** Distribution of the allelic variants of vernalization and photoperiod sensitivity genes identified in the durum wheat collection.

Trait	Gene	Allele	Response	Number of genotypes		Frequency (%)	
				Landraces	Modern	Landraces	Modern
Vernalization	*Vrn-A1*	*vrn-A1*	Winter	2	0	1	0
		*Vrn-A1b*	Spring	72	1	48	5
		*Vrn-A1c*	Spring	67	19	44	95
		*Vrn-A1d*	Spring	10	0	7	0
	*Vrn-B1*	*Vrn-B1*	Winter	149	20	99	100
		*Vrn-B1a*	Spring	2	0	1	0
Photoperiod^1)^	*Ppd-A1*	*Ppd-A1b*	Sensitive	119	3	79	15
		*Deletion Cappelle-Desprez*	Sensitive	30	0	20	0
		*GS105 Ppd-A1a*	Insensitive	2	13	1	65
		*GS100 Ppd-A1a*	Insensitive	0	4	0	20
	*Ppd-B1*	*Ppd-B1b*	Sensitive	63	8	42	40
		*Ppd-B1a*	Insensitive	88	12	58	60

*^*1*^Nomenclature described in Wilhelm et al. (2009).*

### Geographical Pattern of *Vrn-1* and *Ppd-1* Alleles

**FIGURE 1:**
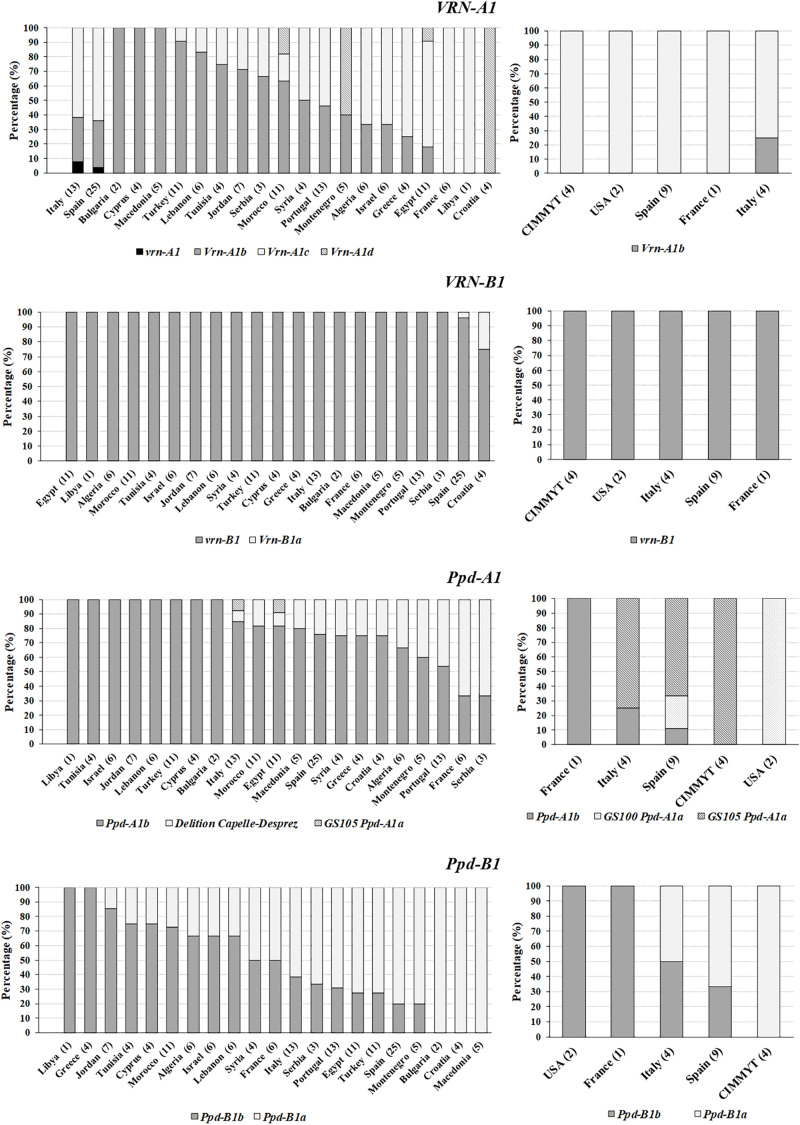
Distribution of the allele variant frequencies (%) for the vernalization and photoperiod sensitivity genes in a collection of durum wheat landraces **(left)** and modern cultivars **(right)** by their geographical origin. Within parentheses the number of entries by each origin.

The most frequent alleles at *Vrn-A1* (*Vrn-A1b* and *Vrn-A1c*), were not evenly geographically distributed (Figure 1). No variability for *Vrn-A1b* was observed in landraces from Bulgaria, Cyprus, and Macedonia, for *Vrn-A1c* in landraces from France and Libya and for *Vrn-A1d* in the Croatian representatives. The allele *vrn-A1* was only present in the Italian landrace ‘Carlantino’ and the Spanish landrace ‘Verdial’ (Supplementary Table S1). All modern cultivars but the Italian ‘Claudio’ were monomorphic for *Vrn-A1c* (Figure 1). The winter allele at *Vrn-B1* predominated in both types of germplasm and only the Croatian landrace ‘Dalmatia 3’ and the Spanish landrace ‘Azulejo de Villa del Río’ carried the spring allele (Supplementary Table S1).

The wild type allele *Ppd-A1b*, causing photoperiod sensitivity, was monomorphic in landraces from Libya, Tunisia, Israel, Jordan, Lebanon, Turkey, Cyprus, and Bulgaria, and predominated in landraces from all other countries except France and Serbia, in which *Ppd-A1*(*DelCD*) was prevalent. Only three modern cultivars carried the sensitive allele *Ppd-A1b*: ‘Arment’ from France, ‘Bolo’ from Spain and ‘Simeto’ from Italy (Supplementary Table S1). Mutation *GS105* was only found in one Egyptian (‘P1-113397’) and one Italian (‘Carlo jucci’) landrace, but it was the only allele of *Ppd-A1* in CIMMYT germplasm and the predominant allele in modern Italian and Spanish cultivars (Figure 1). The mutation *GS100* was present in the two North American cultivars. The allele causing photoperiod insensitivity at *Ppd-B1* was the only one present in landraces from Bulgaria, Croatia and Macedonia and in CIMMYT-derived germplasm, while the sensitive allele of this gene predominated in landraces from most North African countries and in modern French and North American cultivars.

### Effects of Single *Vrn-1* and *Ppd-1* Alleles on Crop Development

The results of the ANOVAs conducted separately for each type of germplasm, growth stage and gene revealed significant differences (*P* < 0.05) in the mean values of years for all growth stages and in the mean values of alleles within each gene for most growth stages (Table 3). The nature of a possible allele variant × year interaction was graphically analyzed for each growth stage, and the results indicated that it was always quantitative in nature, as the order ranks recorded during the 6 years of experiments were maintained (data for GS65 in Supplementary Figure S2). Differences between allele variants increased from the beginning of jointing onwards (Table 3). The four alleles found at *Vrn-A1* in landraces were clustered into three groups according to their effect on the number of days needed to reach the different growth stages. The allele *Vrn-A1d*, found in ten landraces, resulted in a similar phenological development to that of the winter allele found in two of them, while the allele *Vrn-A1b* shortened the developmental pattern by about 2 days at heading and flowering, and *Vrn-A1c* still shortened it by about one more day (Table 3). In the present group of modern cultivars, differences in the number of days needed to reach the various growth stages between the alleles *Vrn-A1b* and *Vrn-A1c* were only statistically significant at GS45. The two alleles found at *Vrn-B1* in landraces resulted in a similar pattern of development, while all the modern cultivars included in the present study were monomorphic for the winter allele at *Vrn-B1*.

**TABLE 3:** Average number of days ± SE from sowing to GS31 (beginning of jointing), GS33 (mid-jointing), GS45 (booting), GS55 (heading), GS65 (anthesis), and GS87 (physiological maturity) for each allele variant of *Vrn-1* and *Ppd-1* genes for 151 durum wheat landraces and 20 modern cultivars.

Gene	*Allele*	Response	*N*	GS31	GS33	GS45	GS55	GS65	GS87
**Lamdraces**									
*Vrn-A1*	*vrn-A1*	Winter	2	126.7 ± 0.87^a^	142.2 ± 2.02^ab^	152.7 ± 0.97^a^	156.6 ± 1.62^a^	161.3 ± 1.88^a^	193.0 ± 2.60^a^
	*Vrn-A1b*	Spring	72	125.1 ± 0.23^a^	140.4 ± 0.30^b^	150.5 ± 0.28^b^	153.9 ± 0.32^b^	159.4 ± 0.34^b^	191.5 ± 0.46^b^
	*Vrn-A1c*	Spring	67	124.4 ± 0.21^a^	139.8 ± 0.31^c^	149.5 ± 0.27^c^	153.1 ± 0.34^c^	158.6 ± 0.35^c^	190.7 ± 0.49^c^
	*Vrn-A1d*	Spring	10	126.2 ± 0.53^a^	142.2 ± 0.96^a^	153.6 ± 0.64^a^	156.6 ± 0.77^a^	162.0 ± 0.86^a^	193.5 ± 1.20^a^
*Vrn-B1*	*vrn-B1*	Winter	149	124.9 ± 0.15^a^	140.3 ± 0.21^a^	150.2 ± 0.19^a^	153.7 ± 0.22^a^	159.2 ± 0.24^a^	191.3 ± 0.32^a^
	*Vrn-B1a*	Spring	2	123.7 ± 1.83^a^	141.6 ± 2.40^a^	152.7 ± 2.39^a^	155.5 ± 2.26^a^	160.7 ± 2.32^a^	192.2 ± 2.92^a^
*Ppd-A1*	*Del CD*	Sensitive	30	125.7 ± 0.29^a^	141.4 ± 0.53^a^	152.6 ± 0.32^a^	156.8 ± 0.42^a^	161.9 ± 0.48^a^	193.1 ± 0.66^a^
	*Ppd-A1b*	Sensitive	119	124.7 ± 0.17^b^	140.1 ± 0.23^b^	149.8 ± 0.22**^b^**	153.0 ± 0.25^b^	158.7 ± 0.26^b^	190.9 ± 0.36^b^
	*GS105 Ppd-A1a*	Insensitive	2	123.0 ± 0.88^b^	137.4 ± 1.31^c^	145.5 ± 0.33**^c^**	148.3 ± 1.44^c^	154.1 ± 1.81^c^	188.8 ± 2.92^c^
*Ppd-B1*	*Ppd-B1b*	Sensitive	63	124.2 ± 0.21^b^	139.5 ± 0.30^b^	148.9 ± 0.28^b^	152.3 ± 0.34^b^	158.1 ± 0.36^b^	190.8 ± 0.50^b^
	*Ppd-B1a*	Insensitive	88	125.4 ± 0.20^a^	140.9 ± 0.29^a^	151.2 ± 0.24^a^	154.7 ± 0.28^a^	160.1 ± 0.31^a^	191.7 ± 0.41^a^
**Modern**									
*Vrn-A1*	*Vrn-A1b*	Spring	1	123.8 ± 0.63^a^	138.9 ± 1.83^a^	146.8 ± 0.49^a^	147.8 ± 2.37^a^	154.7 ± 2.76^a^	189.8 ± 4.58^a^
	*Vrn-A1c*	Spring	19	124.3 ± 0.30^a^	137.7 ± 0.33^a^	144.8 ± 0.25^b^	146.5 ± 0.52^a^	153.8 ± 0.59^a^	188.9 ± 0.95^a^
*Vrn-B1*	*vrn-B1*	Winter	20	124.2 ± 0.29	137.8 ± 0.33	144.9 ± 0.24	146.6 ± 0.50	153.8 ± 0.58	188.9 ± 0.92
*Ppd-A1*	*Ppd-A1b*	Sensitive	3	124.7 ± 0.45^a^	138.4 ± 0.71^a^	146.1 ± 0.59^a^	147.6 ± 1.38^a^	154.9 ± 1.59^a^	190.2 ± 2.35^a^
	*GS105 Ppd-A1a*	Insensitive	13	124.3 ± 0.30^a^	137.9 ± 0.43^a^	144.8 ± 0.28^a^	146.8 ± 0.61^b^	153.9 ± 0.70^b^	188.8 ± 1.16^b^
	*GS100 Ppd-A1a*	Insensitive	4	123.6 ± 1.02^a^	137.1 ± 0.67^a^	143.9 ± 0.56^a^	145.0 ± 1.16^c^	152.7 ± 1.34^c^	188.3 ± 2.11^b^
*Ppd-B1*	*Ppd-B1b*	Sensitive	8	123.6 ± 0.56^b^	137.4 ± 0.49^a^	144.5 ± 0.38^a^	145.8 ± 0.82^b^	153.3 ± 0.95^b^	188.6 ± 1.46^a^
	*Ppd-B1a*	Insensitive	12	124.7 ± 0.29^a^	138.1 ± 0.44^a^	145.1 ± 0.31^a^	147.1 ± 0.63^a^	154.2 ± 0.72^a^	189.2 ± 1.20^a^

*Data from GS31 to GS45 are means across 3 years and those from GS55 to GS87 are means across 6 years. Means within genes for each type of germplasm with the same letters are not significantly different for a Tukey test at *P* < 0.05.*

In terms of photoperiod response, *DelCD* at *Ppd-A1* consistently extended the cycle length from the beginning of jointing to maturity in landraces when compared with the wild type (Table 3). In contrast, the insensitive mutation *GS105* resulted in a reduction of about 4 days to anthesis compared to the wild type. In the present set of modern cultivars, the shortest cycle length was consistently caused by allele *GS100*, with *GS105* showing a lower but statistically significant effect on shortening of crop development from heading. Unexpectedly, the allele *Ppd-B1a* causing photoperiod insensitivity delayed crop development compared with the wild type, in both the landraces and the modern cultivars.

### Effects of Allele Combinations at *Vrn-1* on Crop Development

The effects of the *Vrn-A1* + *Vrn-B1* allele combinations on the six growth stages were examined in both tested panels. The results of the joint ANOVA revealed a significant year effect (*P* < 0.05) in the number of days needed to reach each growth stage, while the plots of the *Vrn-1* allele combinations × year interaction showed that for all growth stages the interaction was quantitative (non-cross-over) in nature (data for GS65 in Supplementary Figure S3A). In total, there were six and two different allele combinations in the landraces and modern cultivars, respectively. Among them, the most frequent allele combinations were *Vrn-A1c* + *vrn-B1*, present in 44% of the landraces and 95% of the modern cultivars, and *Vrn-A1b* + *vrn-B1*, present in 47% of the landraces and 5% of the modern cultivars (Table 4). Both combinations led to a similar pattern of development in the two types of germplasm. In the landraces, both combinations led to a significantly shorter pattern of development than that of the landrace harboring allele combination *Vrn-A1d* + *Vrn-B1a*, which was the latest. The only landrace harboring allele combination *Vrn-A1b* + *VrnB1a* resulted in the shortest developmental pattern. The two landraces harboring double-recessive *vrn-A1* + *vrn-B1* alleles resulting in a winter growth habit led to a similar phenological development to that of the nine landraces carrying combination *Vrn-A1d* + *vrn-B1*, resulting in both cases in an intermediate cycle length (Table 4).

**TABLE 4:** Average number of days ± SE from sowing to GS31 (beginning of jointing), GS33 (mid-jointing), GS45 (booting), GS55 (heading), GS65 (anthesis), and GS87 (physiological maturity) for allele combinations of *Vrn-1* genes identified in 151 durum wheat landraces and 20 modern cultivars.

*Vrn-A1* allele	*Vrn-B1* allele	N	GS31	GS33	GS45	GS55	GS65	GS87
**Landraces**								
*vrn-A1*	*vrn-B1*	2	126.7 ± 0.87^a^	142.2 ± 2.02^ab^	152.7 ± 0.97^bc^	156.6 ± 1.62^b^	161.3 ± 1.88^b^	193.0 ± 2.60^b^
*Vrn-A1b*	*vrn-B1*	71	125.2 ± 0.23^a^	140.4 ± 0.30^b^	150.5 ± 0.28^cd^	153.9 ± 0.32^c^	159.4 ± 0.34^c^	191.5 ± 0.46^bc^
*Vrn-A1b*	*Vrn-B1a*	1	121.1 ± 1.25^b^	139.4 ± 2.50^b^	148.0 ± 1.73^e^	151.2 ± 3.18^d^	157.5 ± 3.56^d^	189.1 ± 4.61^d^
*Vrn-A1c*	*vrn-B1*	67	124.4 ± 0.21^ab^	139.8 ± 0.31^b^	149.5 ± 0.27^de^	153.1 ± 0.34^c^	158.6 ± 0.35^cd^	190.7 ± 0.49^cd^
*Vrn-A1d*	*vrn-B1*	9	126.2 ± 0.52^a^	142.0 ± 0.99^ab^	153.2 ± 0.64^b^	156.2 ± 0.81^b^	161.8 ± 0.92^b^	193.3 ± 1.28^b^
*Vrn-A1d*	*Vrn-B1a*	1	126.3 ± 2.91^a^	143.8 ± 4.23^a^	157.4 ± 1.76^a^	159.7 ± 2.26^a^	164.0 ± 2.61^a^	195.3 ± 3.54^a^
**Modern**								
*Vrn-A1b*	*vrn-B1*	1	123.8 ± 0.63^a^	138.9 ± 1.83^a^	146.8 ± 0.49^a^	147.8 ± 2.37^a^	154.7 ± 2.76^a^	189.8 ± 4.58^a^
*Vrn-A1c*	*vrn-B1*	19	124.3 ± 0.30^a^	137.7 ± 0.33^a^	144.8 ± 0.25^a^	146.5 ± 0.52^a^	153.8 ± 0.59^a^	188.9 ± 0.95^a^

**Data from GS31 to GS45 are means across 3 years and those from GS55 to GS87 are means across 6 years. Means within columns for each type of germplasm with the same letters are not significantly different for a Tukey test at P < 0.05.**

### Effects of Allele Combinations at *Ppd-1* on Crop Development

We also assessed the effects of the *Ppd-A1* + *Ppd-B1* allele combinations on the pattern of development of the landraces and modern cultivars. The plots of the *Ppd-1* × year interaction revealed that although the number of days to reach each growth stage differed significantly between years (*P* < 0.05), the ranking of the *Ppd-A1* + *Ppd-B1* allele combinations was stable across years (data for GS65 in Supplementary Figure S3B). The presence of *DelCD* at *Ppd-A1* in the landraces resulted in the longest cycle length independently of the allele variant present at *Ppd-B1* (Table 5). Different results were obtained in the landraces and modern cultivars regarding the combinations harboring the allele causing photoperiod sensitivity at *Ppd-A1*. In the modern cultivars, the two combinations carrying allele *Ppd-A1b* resulted in a similar number of days to all growth stages. However, in the landraces the combination carrying the allele *Ppd-A1b* and the allele causing photoperiod insensitivity at *Ppd-B1* resulted in a longer cycle length from booting to maturity than the combination in which alleles causing photoperiod sensitivity were present in both genes (Table 5). In the landraces, the only combination carrying alleles causing photoperiod insensitivity at both genes resulted in the shortest cycle length.

**TABLE 5:** Average number of days ± SE from sowing to GS31 (beginning of jointing), GS33 (mid-jointing), GS45 (booting), GS55 (heading), GS65 (anthesis), and GS87 (physiological maturity) for allele combinations of *Ppd-1* genes identified in 151 durum wheat landraces and 20 modern cultivars.

*Ppd-A1* allele	*Ppd-B1* allele	N	GS31	GS33	GS45	GS55	GS65	GS87
**Landraces**								
*Del CD*	*Ppd-B1b*	11	125.4 ± 0.52^ab^	141.3 ± 0.86^a^	152.2 ± 0.53^ab^	156.7 ± 0.67^a^	161.5 ± 0.76^a^	193.2 ± 1.07^a^
*Del CD*	*Ppd-B1a*	19	125.8 ± 0.35^a^	141.4 ± 0.68^a^	152.9 ± 0.40^a^	156.9 ± 0.54^a^	162.1 ± 0.61^a^	193.0 ± 0.84^a^
*Ppd-A1b*	*Ppd-B1a*	67	125.4 ± 0.24^ab^	140.8 ± 0.32^ab^	151.0 ± 0.28^b^	154.3 ± 0.33^b^	159.7 ± 0.35^b^	191.4 ± 0.47^b^
*Ppd-A1b*	*Ppd-B1b*	52	123.9 ± 0.23^bc^	139.1 ± 0.31^bc^	148.2 ± 0.29^c^	151.4 ± 0.37^c^	157.4 ± 0.39^c^	190.3 ± 0.56^c^
*GS105*	*Ppd-B1a*	2	123.0 ± 0.88^c^	137.4 ± 1.31^c^	145.5 ± 0.33^d^	148.3 ± 1.44^d^	154.1 ± 1.81^d^	188.8 ± 2.92^d^
**Modern**								
*Ppd-A1b*	*Ppd-B1b*	1	124.5 ± 0.99^a^	138.2 ± 1.06^a^	145.8 ± 0.75^a^	147.8 ± 2.66^a^	155.1 ± 3.07^a^	190.4 ± 4.06^a^
*Ppd-A1b*	*Ppd-B1a*	2	124.8 ± 0.53^a^	138.5 ± 1.00^a^	146.3 ± 0.83^a^	147.5 ± 1.68^a^	154.8 ± 1.93^a^	190.1 ± 3.00^a^
*GS105*	*Ppd-B1a*	10	124.6 ± 0.33^a^	138.0 ± 0.49^a^	144.8 ± 0.32^a^	147.0 ± 0.69^ab^	154.0 ± 0.78^ab^	189.0 ± 1.32^ab^
*GS105*	*Ppd-B1b*	3	123.3 ± 0.63^a^	137.6 ± 0.93^a^	144.9 ± 0.60^a^	146.1 ± 1.31^bc^	153.5 ± 1.58^bc^	188.2 ± 2.45^b^
*GS100*	*Ppd-B1b*	4	123.6 ± 1.02^a^	137.1 ± 0.67^a^	143.9 ± 0.56^a^	145.0 ± 1.16^c^	152.7 ± 1.34^c^	188.3 ± 2.11^b^

**Data from GS31 to GS45 are means across 3 years and those from GS55 to GS87 are means across 6 years. Means within columns for each type of germplasm with the same letters are not significantly different for a Tukey test at P < 0.05.**

In the modern cultivars, allele *Ppd-A1a* (*GS105*) tended to reduce time to flowering compared with allele *Ppd-A1b*, and its effect was similar independently of the allele variant present at *Ppd-B1*. However, allele *Ppd-A1a* (*GS100*) had the greatest effect on shortening cycle length (Table 5).

### Interactive Effects of *Vrn-1* + *Ppd-1* Allele Combinations on Crop Development

For a comprehensive perspective, we also examined and assessed the interactive effects of the *Vrn-1* + *Ppd-1* allele combinations, even though we recognize that the complex genetic background and the different sample sizes would prevent us from providing definitive results. Sixteen *Vrn-1* + *Ppd-1* allele combinations (ACs) were found in the landraces and six in the modern varieties, with only three being common to both germplasm panels (AC-6, AC-8, and AC-18) (Table 6). The three most frequent combinations in landraces (AC-5, AC-8, and AC-9) were found in 23, 22, and 18% of genotypes, respectively. They contained the alleles *Vrn-A1b* and *Vrn-A1c* at *Vrn-A1*, were monomorphic at *vrn-B1* and *Ppd-A1b* and included the two alleles at *Ppd-B1*. The most common combination in the present group of modern cultivars (AC-18) [*Vrn-A1c* + *vrn-B1* + *Ppd-A1a* (*GS105*) + *Ppd-B1a*] was present in 50% of them, but only in two landraces, the Italian ‘Carlo jucci’ and the Egyptian ‘IP1’ (Supplementary Table S1).

**TABLE 6:** Average number of days ± SE from sowing to GS31 (beginning of jointing), GS33 (mid-jointing), GS45 (booting), GS55 (heading), GS65 (anthesis), and GS87 (physiological maturity) for *Vrn-1* + *Ppd-1* allele combinations identified in 151 landraces and 20 modern cultivars.

Allele combination number	*Vrn-A1* allele	*Vrn-B1* allele	*Ppd-A1* allele	*Ppd-B1* allele	N	GS31	GS33	GS45	GS55	GS65	GS87
**Landraces**											
AC-4	*Vrn-A1d*	*Vrn-B1a*	*Del CD*	*Ppd-B1a*	1	126.3 ± 2.91^ab^	143.8 ± 4.23^a^	157.4 ± 1.76^a^	159.7 ± 2.26^a^	164.0 ± 2.61^a^	195.3 ± 3.54^a^
AC-15	*Vrn-A1d*	*vrn-B1*	*Del CD*	*Ppd-B1a*	2	125.6 ± 1.19^ab^	141.7 ± 2.65^abc^	153.8 ± 1.14^b^	157.6 ± 1.80^ab^	164.0 ± 1.93^a^	193.8 ± 2.72^ab^
AC-14	*Vrn-A1b*	*vrn-B1*	*Del CD*	*Ppd-B1a*	7	126.3 ± 0.62^ab^	141.8 ± 1.10^abc^	153.8 ± 0.49^b^	157.6 ± 0.87^ab^	162.9 ± 0.96^ab^	193.6 ± 1.32^ab^
AC-1	*vrn-A1*	*vrn-B1*	*Ppd-A1b*	*Ppd-B1b*	1	127.6 ± 1.08^a^	142.8 ± 2.70^ab^	153.8 ± 0.95^b^	157.6 ± 2.38^ab^	162.4 ± 2.54^abc^	193.4 ± 3.64^ab^
AC-11	*Vrn-A1c*	*vrn-B1*	*Del CD*	*Ppd-B1b*	9	125.7 ± 0.55^ab^	141.3 ± 0.95^abcd^	152.3 ± 0.62^bc^	157.0 ± 0.74^bc^	161.8 ± 0.83^bcd^	193.4 ± 1.18^ab^
AC-10	*Vrn-A1d*	*vrn-B1*	*Ppd-A1b*	*Ppd-B1a*	5	126.5 ± 0.71^ab^	142.3 ± 1.36^ab^	153.3 ± 0.97^b^	156.3 ± 1.11^bcd^	161.6 ± 1.26^bcde^	193.0 ± 1.71^ab^
AC-13	*Vrn-A1c*	*vrn-B1*	*Del CD*	*Ppd-B1a*	9	125.5 ± 0.44^ab^	140.8 ± 0.93^abcd^	151.4 ± 0.54^bcd^	155.8 ± 0.78^bcd^	160.9 ± 0.92^bcde^	192.1 ± 1.27^bc^
AC-9	*Vrn-A1b*	*vrn-B1*	*Ppd-A1b*	*Ppd-B1a*	28	126.3 ± 0.39^ab^	141.5 ± 0.50^abc^	152.2 ± 0.41^bc^	155.4 ± 0.48^cde^	160.7 ± 0.52^cde^	192.2 ± 0.70^bc^
AC-2	*vrn-A1*	*vrn-B1*	*Del CD*	*Ppd-B1b*	1	125.7 ± 1.31^ab^	141.6 ± 3.56^abc^	151.6 ± 1.63^bc^	155.7 ± 2.35^bcd^	160.3 ± 2.94^def^	192.6 ± 4.07^bc^
AC-7	*Vrn-A1d*	*vrn-B1*	*Ppd-A1b*	*Ppd-B1b*	2	125.9 ± 1.12^ab^	141.8 ± 1.76^abc^	152.2 ± 1.14^bc^	154.7 ± 1.53^de^	160.3 ± 1.83^def^	193.6 ± 2.99^ab^
AC-12	*Vrn-A1b*	*vrn-B1*	*Del CD*	*Ppd-B1b*	1	122.6 ± 2.36^bc^	141.0 ± 3.30^abcd^	151.4 ± 1.10^bcd^	155.3 ± 2.17^cde^	159.7 ± 2.77^efg^	192.2 ± 3.85^bc^
AC-8	*Vrn-A1c*	*vrn-B1*	*Ppd-A1b*	*Ppd-B1a*	33	124.5 ± 0.30^abc^	140.0 ± 0.44^abcd^	149.6 ± 0.37^cde^	153.2 ± 0.48^ef^	158.6 ± 0.50^fgh^	190.6 ± 0.70^cd^
AC-5	*Vrn-A1b*	*vrn-B1*	*Ppd-A1b*	*Ppd-B1b*	35	124.1 ± 0.28^abc^	139.3 ± 0.38^bcd^	148.5 ± 0.35^def^	152.0 ± 0.44^f^	157.8 ± 0.47^gh^	190.6 ± 0.68^cd^
AC-3	*Vrn-A1b*	*Vrn-B1a*	*Ppd-A1b*	*Ppd-B1a*	1	121.1 ± 1.25^c^	139.4 ± 2.50^bcd^	148.0 ± 1.73^ef^	151.2 ± 3.18^fg^	157.5 ± 3.56^hi^	189.1 ± 4.61^d^
AC-6	*Vrn-A1c*	*vrn-B1*	*Ppd-A1b*	*Ppd-B1b*	14	123.0 ± 0.42^bc^	138.1 ± 0.55^cd^	146.8 ± 0.51^ef^	149.1 ± 0.70^gh^	155.7 ± 0.75^ij^	188.7 ± 1.12^d^
AC-18	*Vrn-A1c*	*vrn-B1*	*GS105*	*Ppd-B1a*	2	123.0 ± 0.88^bc^	137.4 ± 1.31^d^	145.5 ± 0.33^f^	148.3 ± 1.44^h^	154.1 ± 1.81^j^	188.8 ± 2.92^d^
**Modern**											
AC-6	*Vrn-A1c*	*vrn-B1*	*Ppd-A1b*	*Ppd-B1b*	1	124.5 ± 0.99^a^	138.2 ± 1.06^a^	145.8 ± 0.75^a^	147.8 ± 2.66^a^	155.1 ± 3.07^a^	190.4 ± 4.06^a^
AC-8	*Vrn-A1c*	*vrn-B1*	*Ppd-A1b*	*Ppd-B1a*	2	124.8 ± 0.53^a^	138.5 ± 1.00^a^	146.3 ± 0.83^a^	147.5 ± 1.68^a^	154.8 ± 1.93^a^	190.1 ± 3.00^a^
AC-17	*Vrn-A1b*	*vrn-B1*	*GS105*	*Ppd-B1b*	1	123.8 ± 0.63^a^	138.9 ± 1.83^a^	146.8 ± 0.49^a^	147.8 ± 2.37^a^	154.7 ± 2.76^a^	189.8 ± 4.58^ab^
AC-18	*Vrn-A1c*	*vrn-B1*	*GS105*	*Ppd-B1a*	10	124.6 ± 0.33^a^	138.0 ± 0.49^a^	144.8 ± 0.32^a^	147.0 ± 0.69^a^	154.0 ± 0.78^ab^	189.0 ± 1.32^ab^
AC-16	*Vrn-A1c*	*vrn-B1*	*GS105*	*Ppd-B1b*	2	123.1 ± 0.92^a^	137.0 ± 1.08^a^	143.9 ± 0.48^a^	145.2 ± 1.58^b^	152.8 ± 1.98^bc^	187.4 ± 2.99^c^
AC-19	*Vrn-A1c*	*vrn-B1*	*GS100*	*Ppd-B1b*	4	123.6 ± 1.02^a^	137.1 ± 0.67^a^	143.9 ± 0.56^a^	145.0 ± 1.16^b^	152.7 ± 1.34^c^	188.3 ± 2.11^bc^

*Combinations are arranged from the longest to the shortest time to anthesis. Data from GS31 to GS45 are means across 3 years and those from GS55 to GS87 are means across 6 years. Means within columns for each type of germplasm with the same letters are not significantly different for a Tukey test at *P* < 0.05.*

As in previous ANOVAs, the year effect accounted for most of the phenotypic variance during crop developmental stages. However, the *Vrn-1* + *Ppd-1* allele combination × year interaction was non-crossover in nature in all them (data for GS65 in Supplementary Figure S3C). Regarding landraces, the ranges of the mean number of days to reach a given growth stage were lower at GS31 (6 days), GS33 (7 days), and GS87 (7 days) than at GS45 (12 days), GS55 (11 days), and GS65 (10 days) (Table 6). Although many combinations led to developmental patterns without statistically significant differences, it can be seen in Table 6 that the combinations harboring the alleles *vrn-A1* + *vrn-B1*, associated with a winter growth habit, did not lead to the longest cycle. The comparison of the cycle length of the two landraces harboring the recessive alleles at the two genes (*vrn-A1* + *vrn-B1*, AC-1, and AC-2), which only differed in the allele variant at *Ppd-A1* [*Ppd-A1b* in one and *Ppd-A1*(*DelCD*) in the other], revealed that the presence of *Ppd-A1*(*DelCD*) reduced the time to anthesis by about 2 days. However, the comparison of combinations only differing in the *Ppd-A1* allele (AC-14 with AC-9, AC-13 with AC-8, AC-15 with AC-10, AC-12 with AC-5 and AC-11 with AC-6) showed that the allele *Ppd-A1*(*DelCD*) consistently extended the phenological development of the crop. It is also noteworthy that the three allele combinations at the top of Table 6, showing the longest cycle length at anthesis, had the allele *Ppd-A1*(*DelCD*).

The effect of the allele variant at *Ppd-B1* when comparing allele combinations with the same alleles at the other loci (AC-14 with AC-12, AC-10 with AC-7, AC-9 with AC-5 and AC-8 with AC-6) confirmed the developmental delay caused by the allele *Ppd-B1a*. In the landraces the only combination carrying the mutation *GS105* at *Ppd-A1* causing photoperiod insensitivity resulted in the earliest phenotypes consistently across growth stages.

The effect of the *Vrn-1* + *Ppd-1* allele combinations identified in the modern cultivars was not noticeable before heading (bottom part of Table 6). The comparison of ACs differing only in the allele at *Vrn-A1* (AC-17 and AC-16) found in one and two landraces, respectively, showed that the presence of allele *b* at this gene enlarged the cycle length by 2 days in comparison with allele *c*. Allele combinations carrying *Ppd-A1b* resulted in the longest developmental pattern, followed by those harboring the mutations *GS105* and *GS100* (Table 6). Allele variants at *Ppd-A1* with the same alleles at the other three loci (comparisons AC-8 with AC-18 and AC-16 with AC-19) led to similar patterns of development. Differences caused by the presence of the two alleles at *Ppd-B1* were inconsistent because AC-6 led to a similar phenology to that of AC-8, but the presence of allele *a* at this locus significantly delayed development at GS55 and GS87 when AC-16 was compared with AC-18. This confirms the interaction between the *Vrn-1* and *Ppd-1* genes.

## Discussion

Although durum wheat is a major crop in Mediterranean-type climates, the variability existing in the species for the vernalization and photoperiod genes has been poorly analyzed. In the present study, we used DNA markers to assess the allelic variation at the *Vrn-A1*, *Vrn-B1*, *Ppd-A1*, and *Ppd-B1* genes in a collection of ancient durum landraces, using also a set of locally adapted modern cultivars for comparison. We have examined the effect of individual alleles and their allelic combinations on their pattern of development under field conditions during six crop seasons. The use of germplasm pools representative of two breeding eras (landraces grown in the Mediterranean Basin before the ‘Green Revolution’ and locally adapted modern semi-dwarf improved cultivars) allowed us to test the correspondence between ancient and recent genetic backgrounds regarding growth habit and photoperiod response and the variation in crop development.

### Breeding Effects on the Pattern of Development of Durum Wheat

The analysis of the developmental patterns within the collection of durum wheat landraces and the set of locally adapted modern cultivars used in the present study showed that cycle length was shortened as a consequence of breeding. This reduction was noticeable from early growth stages, but was maximized at heading, which took place on average 7 days earlier in the modern cultivars. This result is in line with previous studies reporting a shortening of about 2 and 9 days in the time to anthesis of Italian and Spanish durum wheat varieties, respectively, as a result of breeding activities during the 20th century (Álvaro et al., 2008; Royo et al., 2008; Isidro et al., 2011). Earliness in reaching anthesis was an attribute selected for by breeding programs to enhance the adaptation of improved varieties to environments where springs have an increasing pattern of drought and temperature, such as the one prevalent in the Mediterranean Basin, thus allowing grain to fill under more favorable environmental conditions (Royo et al., 2006).

Only three of the 16 allelic combinations present in landraces were also identified in the modern panel, which incorporated another three combinations not present in the old germplasm. For the three combinations common to both germplasm types, 32% of the landraces harboring them were among the earliest, but 65% of the modern cultivars carrying them were among the latest. This result reflects the efforts undertaken by the breeding programs represented here to release early varieties that can escape from terminal stresses, which was mainly achieved through the introgression of the two mutations causing photoperiod insensitivity at the *Ppd-A1* gene.

### *Vrn-1* and *Ppd-1* Allele Frequency and Geographic Distribution

The *Vrn-A1* gene in the present landrace collection was mono-allelic (fixed) in only six out of the 21 Mediterranean source countries, with landraces from the reaming 15 countries exhibiting two or three different alleles at this locus. However, a geographic pattern for the allelic distribution at this gene was not apparent. One of the greatest differences in allelic frequencies between the landraces and the modern cultivars included in this study was detected for the *Vrn-A1* gene, as four allele variants were identified in the landraces, but only two in the modern cultivars. The winter allele *vrn-A1* was only identified in two landraces, but it was not present in the present set of modern cultivars. This result is in agreement with the clear preponderance of spring dominant alleles at the *Vrn-A1* gene found previously in improved durum wheat varieties from Argentina, CIMMYT, France, Italy, and Hungary (Basualdo et al., 2015). The allele *Vrn-A1a*, found previously in durum wheat varieties from Russia (Muterko et al., 2016b) was not present in the germplasm analyzed here. In our study, all modern varieties except *cv* ‘Claudio’ carried the spring allele *Vrn-A1c*, while the allele *Vrn-A1b* was present in about half of the landraces, thus suggesting that breeding programs represented here have selected for the allele *Vrn-A1c* against *Vrn-A1b*. This inference agrees with the low frequency of the *Vrn-A1b* allele reported previously in cultivated tetraploid wheat (Yan et al., 2004) and the lack of significant marker-trait association in the chromosomal region corresponding to the *Vrn-A1* locus observed in a genome-wide association panel of 328 modern diverse European genotypes, indicating that the alleles at the *Vrn-A1* gene move toward fixation (Würschum et al., 2019). The *Vrn-A1b allele* was completely absent in a recent collection of 276 ICARDA lines and was found in only 4 of 2,529 genotypes from the current CIMMYT program (Dreisigacker and Ammar, 2016, personal communication), confirming its elimination from the programs of two of the most important germplasm providers worldwide. The reason for this definitive elimination of *Vrn-A1b* from most modern germplasm is unclear and cannot be attributed to its effect on phenology alone. This could not be demonstrated using data of the modern cultivars included in this study because of the rarity of this allele (1 in 20). However, in the landraces group where allelic frequencies of *Vrn-A1b* and *Vrn-A1c* were very similar (48 and 44%, respectively), the delay in phenology associated with the former allele was too reduced in magnitude (less than 1 day for any growth stage) to reasonably explain the systematic selection against it in the quest for earliness by breeding programs.

The landraces and modern cultivars did not differ greatly in the allelic frequency at *Vrn-B1*, as modern cultivars were monomorphic for the winter allele *vrn-B1*, and it was present in all except two landraces. It is well known that the ancestors of wheat were winter types, so they harbored the recessive alleles at *Vrn-A1* and *Vrn-B1*. Chu et al. (2011) suggested that the mechanism by which tetraploid wheat evolved from the winter wild type to the spring type was through a mutation on the *Vrn-B1* gene. Our results are not consistent with this hypothesis because the near fixation of the winter allele *vrn-B1* in the two germplasm panels analyzed here suggests that mutations in the *Vrn-A1* gene should have been the genetic basis underlying the spring growth habit of Mediterranean landraces and modern durum cultivars. The dominant allele *Vrn-B1c*, commonly found in durum wheats from Russia and Ukraine, but not from Europe or the United States (Muterko et al., 2016b), was also absent in our germplasm.

All the allele variants previously reported in durum wheat at *Ppd-A1* were found in this study. The prevalence of the sensitive allele *Ppd-A1b* in landraces but its low frequency in modern varieties may reflect the shuttle-breeding strategy first implemented by Norman E. Borlaug in CIMMYT (Mexico) in the 1960s. The selection in early generations in environments with contrasting photoperiods conducted in this international center resulted in a systematic and intensive selection for photoperiod insensitivity that enhanced the wide adaptation of plants and was an important factor in the success of the ‘Green Revolution’ (Royo et al., 2016).

As far as we know, this study identified for the first time in durum wheat the *Ppd-A1*(*DelCD*) allele, which was present in 20% of the landraces from 13 out of the 21 Mediterranean countries. The candidate null allele on chromosome 2A in ‘Capelle-Desprez’ was first reported by Beales et al. (2007) carrying a deletion of part of exon 5, intron 5 and part of exon 6. These mutations are expected to produce a quantitative delay in flowering. However, in an allelic series of BC_2_F_4_ lines in two hexaploid winter wheat backgrounds, the *Ppd-A1*(*DelCD*) allele showed little influence on flowering (Bentley et al., 2013). The effect of the mutations in hexaploid wheat may be masked by the presence of intact genes on the other genomes. In the same study by Bentley et al. (2013) and others, the D-genome photoperiod gene had a more potent effect on flowering than the gene on the B genome. The effect of the *Ppd-A1*(*DelCD*) allele may therefore be more pronounced in durum than in hexaploid bread wheat. However, the identity of this allele from durum landraces to that present in bread wheat should have to be clearly validated by sequence comparison after re-sequencing.

As expected, most landraces that originated in countries located in the Fertile Crescent harbored the wild allele at *Ppd-A1*. However, one of the four Syrian landraces (‘IG-95841’), collected in the Daraa region and kindly supplied by the ICARDA Germplasm Bank, harbored the *Ppd-A1*(*DelCD*) allele, which suggests that this mutation on chromosome 2A may have originated in durum wheat in Syria. The comparison of the climate type of Daraa with those of Deir ez-Zor, Al-Hasakeh and Homs, regions of origin of the Syrian landraces ‘IG-95812’, ‘IG-95847,’ and ‘IG-95931,’ respectively, suggest that the mutation may have been an adaptive mechanism to the environmental conditions. According to the Köppen climate classification, the Daraa region has a ‘cold semi-arid’ climate, while the Syrian regions of Deir ez-Zor, Al-Hasakeh, and Homs have hot desert, semi-arid and hot-summer climates, respectively^3^. Therefore, the delay of crop development caused by the *Ppd-A1*(*DelCD*) allele, which on average retarded the anthesis of the Syrian line harboring it by 7–9 days irrespective the Syrian lines carrying the *Ppd-A1b* allele (data not shown), may reflect the adaptation to low temperatures.

The frequency of the insensitive allele *Ppd-A1a* (*GS105*) in modern cultivars was greater than that of allele *Ppd-A1a* (*GS100*), in agreement with previous reports in durum wheat (Bentley et al., 2011; Royo et al., 2016). Our results support the hypothesis that the mutation resulting in the allele *Ppd-A1a* (*GS105*) preceded the one resulting in the allele *Ppd-A1a* (*GS100*), and for this reason the allele *Ppd-A1a* (*GS105*) was detected in the landraces, but allele *Ppd-A1a* (*GS100*) was found only in modern varieties, as in the study by Bentley et al. (2011).

All Bulgarian, Macedonian, and Croatian and two of the three Serbian landraces harbored the allele *Ppd-B1a*, which caused a delay in the pattern of development. The longest cycle length of Balkan landraces was reported in the study by Soriano et al. (2016). The presence of the allele *Ppd-B1b* is in agreement with the low temperatures characteristic of the main wheat growing areas of northern Balkan countries (Royo et al., 2014). In addition, this finding supports the hypothesis that landraces from northern Balkan countries may have a different origin (Nazco et al., 2012; Soriano et al., 2016), probably the steppes of south Russia and the Volga Region, as suggested by Melnikova et al. (2010).

### Effects of the *Vrn-1* and *Ppd-1* Alleles and Allele Combinations on Crop Development

Although the year effect contributed greatly to explaining the phenotypic variance for the number of days needed to reach each growth stage in both germplasm types, the effect of allele variants of each gene and their combinations was consistent across years, conferring reliability on the results obtained in this study. As expected, the differences between the allele effects on crop development were lower in the earlier growth stages but increased progressively until anthesis and maturity.

Usually, spring cereal cultivars do not require a cold period prior to heading, whereas in winter types it is an essential prerequisite for flowering (Roberts et al., 1988). Because one dominant gene, either *Vrn-A1* or *Vrn-B1*, is sufficient to confer a spring growth habit, it was assumed that the two landraces harboring the *vrn-A1* + *vrn-B1* alleles were the only ones with a winter growth habit. Given that these two landraces flowered normally in the six experiments, we assumed that the cold periods of exposure after sowing were always enough to fulfill their vernalization requirements.

The 11 days of difference in heading resulting in this study from the effect of combinations of vernalization and photoperiod genes are similar to the 10 days previously reported in bread wheat (Kiss et al., 2014), in which the major effect genes are *Vrn-D1* and *Ppd-D1*.

Using the methodology described in Yan et al. (2004), we identified the allele *Vrn-A1d* (GenBank AY616462) in ten landraces from four Mediterranean countries. This allele was first found in three *Triticum turgidum* ssp. *dicoccoides* accessions and was designated by Yan et al. (2004), who assumed it to be associated with a dominant spring growth habit but did not provide sufficient evidence. The examination of the effect of the allele *Vrn-A1d* on the development of landraces carried out in this study demonstrated that it led to a similar pattern of development to the one resulting from the presence of the *vrn-A1* winter allele. However, the samples of landraces harboring the two recessive alleles at *vrn-1* were so limited that phenotypic data could not be statistically representative. Nevertheless, our assumption was supported by the comparison of the number of days elapsed to reach each growth stage in landraces harboring six different *Vrn-A1* + *Vrn-B1* allelic combinations, as the two of them that contained the *Vrn-A1d* allele led to the longest cycle length, similar to or even longer than that observed in the two landraces harboring the double-recessive alleles *vrn-A1* + *vrn-B1*, and thus having a winter growth habit. In addition, although the results of the comparison of the 16 *Vrn-1* + *Ppd-1* allelic combinations found in the landraces were more difficult to interpret, it was noticeable that the number of days to each growth stage was quite similar in combinations carrying the two recessive alleles at *Vrn-1* and those carrying the *Vrn-A1d* allele. Moreover, two of the four allele combinations carrying this allele were in the upper part of Table 6, thus showing the longest cycle length. All these results indicate that the presence of the allele *Vrn-A1d* in durum wheat landraces delayed crop development to a similar extent to that of the winter allele *vrn-A1* (*vrn-A1* = *Vrn-A1d*), thus suggesting that the allele *Vrn-A1d* is probably not associated with a dominant spring growth habit. However, this hypothesis will have to be demonstrated by further experiments conducted in controlled conditions.

In this study, the spring alleles *Vrn-A1b* and *Vrn-A1c* were found in both the landraces and the modern cultivars, and the results indicated that *Vrn-A1b* consistently delayed the time needed to reach growth stages from booting to maturity by about 1 day in both tested panels when compared with *Vrn-A1c*. Thus, *Vrn-A1b* > *Vrn-A1c*. On the other hand, the presence of the alleles *Vrn-A1b* or *Vrn-A1*c resulted in a shorter cycle length, independently of the alleles at *Vrn-B1*, but a strong interaction was found between these alleles and the *Ppd-1* genes, as shown by the results of comparing *Vrn-1* + *Ppd-1* allelic combinations. Comparisons made between *Vrn-A1* + *Vrn-B1* allelic combinations showed an interaction between the two genes, as the spring allele at *Vrn-B1* significantly shortened the cycle in presence of the allele *b* at *Vrn-A1* but extended it when the allele *d* was present at the same gene. These results are in line with those reported in bread wheat by Zhang et al. (2008), who found that the *Vrn-A1* allele was associated with the earliest heading time.

The low frequency of *Vrn-B1* gene found in the current study compared with the preponderance of the ancestral wild-type *vrn-B1* is in agreement with the recently observed on a global collection of tetraploid wheat (Maccaferri et al., 2019).

Concerning photoperiod response, all the previously published allelic combinations in durum wheat at *Ppd-A1* and *Ppd-B1* were present in the germplasm used in this study, and a new allele (*DelCD*) at *Ppd-A1* was found for the first time in durum wheat. Further studies for resequencing this allele will be required to confirm its identity in durum wheat. Our results show that landraces harboring the allele *DelCD* at *Ppd-A1* had a consistently longer cycle length than those carrying the sensitive *Ppd-A1b* allele. The effect of *DelCD* on cycle extension was confirmed when a comparison was made between allele variants at *Ppd-A1*, between allele combinations at *Ppd-A1* + *Ppd-B1*, and also between *Vrn-1* + *Ppd-1* allele combinations. Allele *Ppd-A1*(*DelCD*) consistently extended the development of landraces in presence of a dominant allele *Vrn-A1* and the recessive allele *vrn-B1*, independently of the allele present at *Ppd-B1*. However, despite the small sample size, the comparison of the two landraces harboring the two recessive alleles at *Vrn-1* tended to go in the opposite direction, thus suggesting a functional interaction between the *Vrn-1* and *Ppd-1* genes, as suggested by Distelfeld et al. (2009). The delay in crop development caused by the allele *Ppd-A1*(*DelCD*) in Mediterranean landraces indicates that it may confer photoperiod sensitivity.

The presence of the mutation *GS105* at *Ppd-A1* significantly shortened the time to reach each growth stage in landraces, with cycle reduction being greater (up to 6 days) from booting to anthesis. However, although significant, the earliness caused by the mutation *GS105* in the modern cultivars was lower than that in the landraces. In the present set of modern germplasm, the strongest earliness resulted from the presence of the mutant allele *Ppd-A1a* (*GS100*), in agreement with the findings of Royo et al. (2016). Our results support the stronger effect of the allele conferring photoperiod sensitivity at *Ppd-A1* than that at *Ppd-B1* (*Ppd-A1b* > *Ppd-B1b*), as previously reported in durum wheat by Royo et al. (2016). According to our results, the relative effect of single *Ppd-A1* alleles on extending phenological development can be expressed as *Ppd-A1*(*DelCD*) > *Ppd-A1b* > *Ppd-A1a* (*GS105*) > *Ppd-A1a* (*GS100*). However, the clear individual allele effect at *Ppd-A1* was masked in *Vrn-1* + *Ppd-1* allele combinations due to interactions between both genes.

A marker effect could explain the longer cycle length observed in the landraces and modern cultivars carrying the *Ppd-B1a* insensitive allele than in the ones carrying the sensitive allele *Ppd-B1b*, which was an unexpected result. In the study by Royo et al. (2016), the *Ppd-B1a* insensitive allele, identified using the same molecular marker (*gwm148*), showed an opposite and stronger effect on shortening flowering within 25 modern breeding lines. Tanio and Kato (2007) described a *Ppd-B1a* mutation from the Japanese cultivar ‘Fukuwasekomugi’ that was early flowering. Nishida et al. (2013) characterized a *Ppd-B1a* allele (a 308 bp insertion in the 5′-upstream region) tracing back to the Japanese landrace ‘Shiroboro21,’ additionally suggesting that an allelic series of photoperiod insensitivity mutations exists. However, these casual mutations have been observed to be rare. Instead, *Ppd-B1* was shown to be present in different copy numbers, with higher copy number variants conferring photoperiod insensitivity and resulting in earlier heading. Diaz et al. (2012) reported that *Ppd-B1a* alleles conferring early flowering from the hexaploid bread wheat ‘Chinese Spring,’ ‘Sonora64,’ and ‘Récital’ sources had an increased number of copies of *Ppd-B1*. Würschum et al. (2019) described the same in durum wheat, with two copy number variants present. The higher copy number conferred earlier heading and was more frequent in the heat- and drought-prone countries at lower latitudes. The copy number variants appear to alter gene expression in a similar way to the described mutations, but to a lesser extent. While in all other cases in our studies the markers deployed were functional (so within the gene), the marker we used to determine the *Ppd-B1* alleles was only tightly linked to the gene according to Hanocq et al. (2004), so it remains unclear to which type of polymorphism the marker is related. In comparison with Royo et al. (2016), in this study germplasm with larger genetic diversity was also tested, which could additionally affect the linkage of the marker to the gene.

Our results support the findings in hexaploid wheat of Shaw et al. (2012), who classified the photoperiod-insensitive alleles at *Ppd-A1* and *Ppd-B1* as *Ppd-A1a* > *Ppd-B1a*, when *Ppd-A1a* was the *GS100* durum wheat variant. In this study the effect on crop development of the allele variant present at *Ppd-B1* was stronger when *Ppd-A1b* was present at *Ppd-A1* than when *DelCD* was present, supporting the suggestion that in durum wheat the effect of allele *Ppd-B1a* depends on the allele present at *Ppd-A1* (Royo et al., 2016). Interaction between *Ppd-B1* and *Ppd-D1* genes was also reported in bread wheat by Tanio and Kato (2007). Interactive effects of *Vrn-1* and *Ppd-1* gene combinations were also detected in our research.

The three allelic combinations common to both germplasm sets (AC-6, AC-8, and AC-18, Table 6) were identified in the earliest landraces but in the latest modern cultivars. The greater standard deviations of values observed for AC-6 and AC-8 in the growth stages of modern cultivars compared to those of landraces suggests more plasticity in the modern set than in the ancient wheats. Whereas the allelic diversity at *Vrn* and *Ppd* genes was substantially greater in landraces than in the present set of locally adapted cultivars, it produced phenotypic variability that was more oriented toward delaying development. The much more reduced allelic diversity for *Vrn* + *Ppd* in modern cultivars generated a phenological variability that allowed the identification of much shorter cycle types, arguably providing genotypes that can be more performant under most low latitude environments. Our results suggests that the allelic variants associated with longer cycle length, principally *Vrn-A1d* and *Ppd-A1*(*DelCd*) were selected against during the modern breeding process in favor of allelic variants associated with earliness such as *Vrn-A1c* and *Ppd-A1*(*GS105*), which are by far the most frequent in modern cultivars. Since breeders select directly for earliness, a single trait that is affected by both *Vrn* and *Ppd* genes, it is likely that alleles shortening cycle length at both loci were selected simultaneously in breeding programs. This has been shown to be the case in recent studies conducted with Australian bread wheat (Joukhadar et al., 2019) and Italian durum wheat (Taranto et al., 2020) which have demonstrated that artificial selection involved strong simultaneous selection on specific alleles for vernalization and photoperiod genes.

## Conclusion

The selection performed in most spring wheat breeding programs for low latitude environments since the Green Revolution have reduced the allelic diversity at *Vrn-1* and *Ppd-1* genes from that present in ancient landraces, thus resulting in a narrower developmental scope in modern cultivars. The results of this study suggest that the changes introduced in the two genes included a reduction of 50% in the number of allelic variants at the *Vrn-A1* and *Vrn-B1* genes, and the massive introduction of mutant alleles conferring photoperiod insensitivity at *Ppd-A1*.

The removal in modern cultivars of the two alleles at *Vrn-A1* resulting in longer cycle length (*vrn-A1* and *Vrn-A1d*) can be explained by the need to reduce time to flowering as a mechanism for escaping to terminal drought and excessive late occurring heat. The extremely drastic selection for allele *Vrn-A1c* and against allele *Vrn-A1b* suggested by the results presented here may not be explained by their respective effects on phenology alone. As a single dominant allele at *Vrn-A1* confers a spring growth habit, the replacement of the winter allele at *Vrn-B1* was probably unnecessary to obtain the spring habit needed to be competitive in most Mediterranean environments.

The precise characterization of vernalization- and photoperiod-related genes at molecular level in ancient and modern germplasm and their association with crop development will help to fine-tune the *Vrn-1* and *Ppd-1* gene composition to optimize the adaptation of the new durum wheat varieties to the upcoming climate scenarios.

## Data Availability Statement

The datasets presented in this study can be found in online repositories. The names of the repository/repositories and accession number(s) can be found below: http://hdl.handle.net/11529/10548410.

## Author Contributions

CR, KA, and DV conceived the project. CR and DV assembled and purified the germplasm collection and performed field evaluations. SD performed molecular analyses. CR carried out the statistical analyses and outlined the manuscript. All authors wrote the manuscript and approved the final version.

## Conflict of Interest

The authors declare that the research was conducted in the absence of any commercial or financial relationships that could be construed as a potential conflict of interest.

## Footnotes

1 http://www.cites.org

2 www.lgcgenomics.com

3 http://hanschen.org/koppen/

## Supplementary Material

FIGURE S1 Maximum, mean and minimum daily air temperature and the variation in day length from physiological maturity over the six years of field experiments.

FIGURE S2 Individual alleles at each gene × year interaction for the number of days to GS65 (anthesis) in landraces **(left)** and modern cultivars **(right)**.

FIGURE S3 Allele combinations × year interaction for the number of days to GS65 (anthesis) in landraces (left) and modern cultivars (right). **(A)** Allele combinations of *Vrn-1* genes; **(B)** allele combinations of *Ppd-1* genes; **(C)** allele combinations of *Vrn-1* + *Ppd-1* genes. See Table 6 for the meaning of allele combination (AC) numbers.

TABLE S1 Allelic variants of vernalization and photoperiod sensitivity genes on each genotype of the durum wheat collection studied.

TABLE S2 Allelic variants of vernalization and photoperiod sensitivity genes observed in durum wheat.

TABLE S3 Marker summary.
