# Supplementary material for: Allelic Variation at the Vernalization Response (Vrn-1) and Photoperiod Sensitivity (Ppd-1) Genes and Their Association With the Development of Durum Wheat Landraces and Modern Cultivars
Source: Front Plant Sci. 2020 Jun 23;11:838. doi: 10.3389/fpls.2020.00838 (PMC7325763; doi:10.3389/fpls.2020.00838)
Supplement: FIGURE S1 — Maximum, mean and minimum daily air temperature and the variation in day length from physiological maturity over the six years of field experiments. [file Image_1.pdf]

SUPPLEMENTARY FIGURE S1. Maximum, mean and minimum daily air temperature and the variation in day length from physiological maturity over the six years of field experiments.

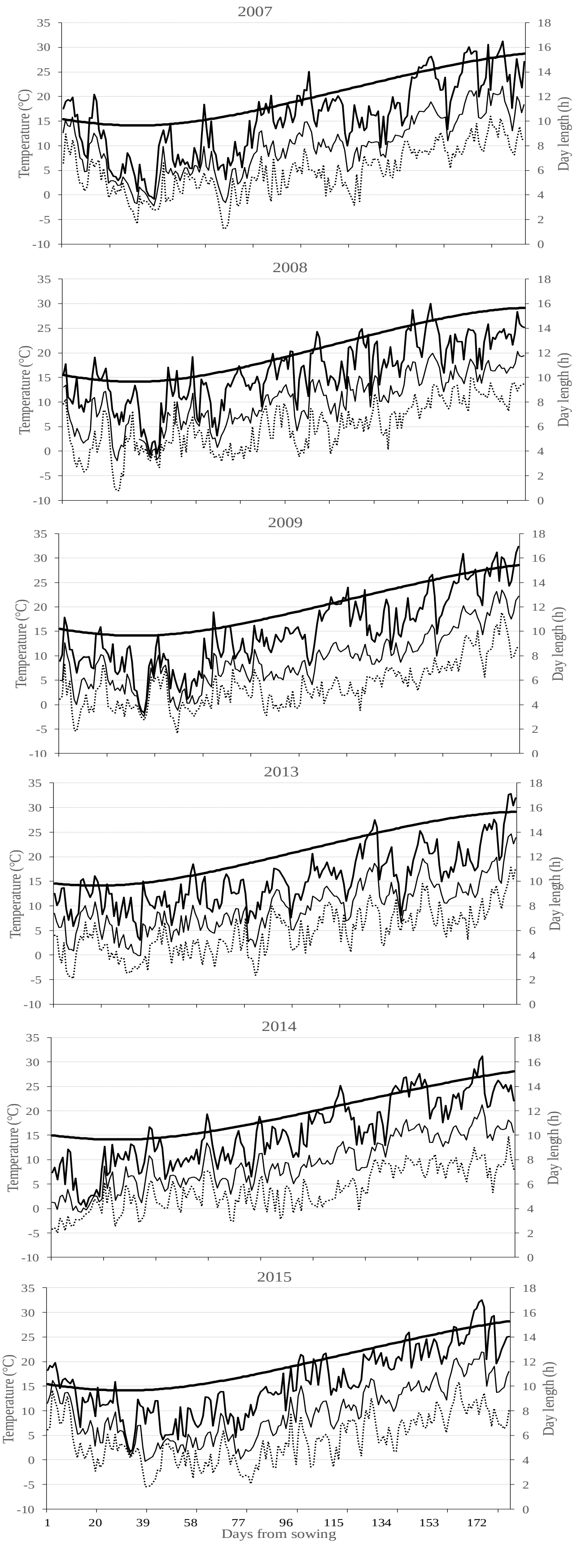

.
