# Supplementary material for: Allelic Variation at the Vernalization Response (Vrn-1) and Photoperiod Sensitivity (Ppd-1) Genes and Their Association With the Development of Durum Wheat Landraces and Modern Cultivars
Source: Front Plant Sci. 2020 Jun 23;11:838. doi: 10.3389/fpls.2020.00838 (PMC7325763; doi:10.3389/fpls.2020.00838)

SUPPLEMENTARY FIGURE S2. Individual alleles at each gene × year interaction for the number of days to GS65 (anthesis) in landraces (left) and modern cultivars (right).

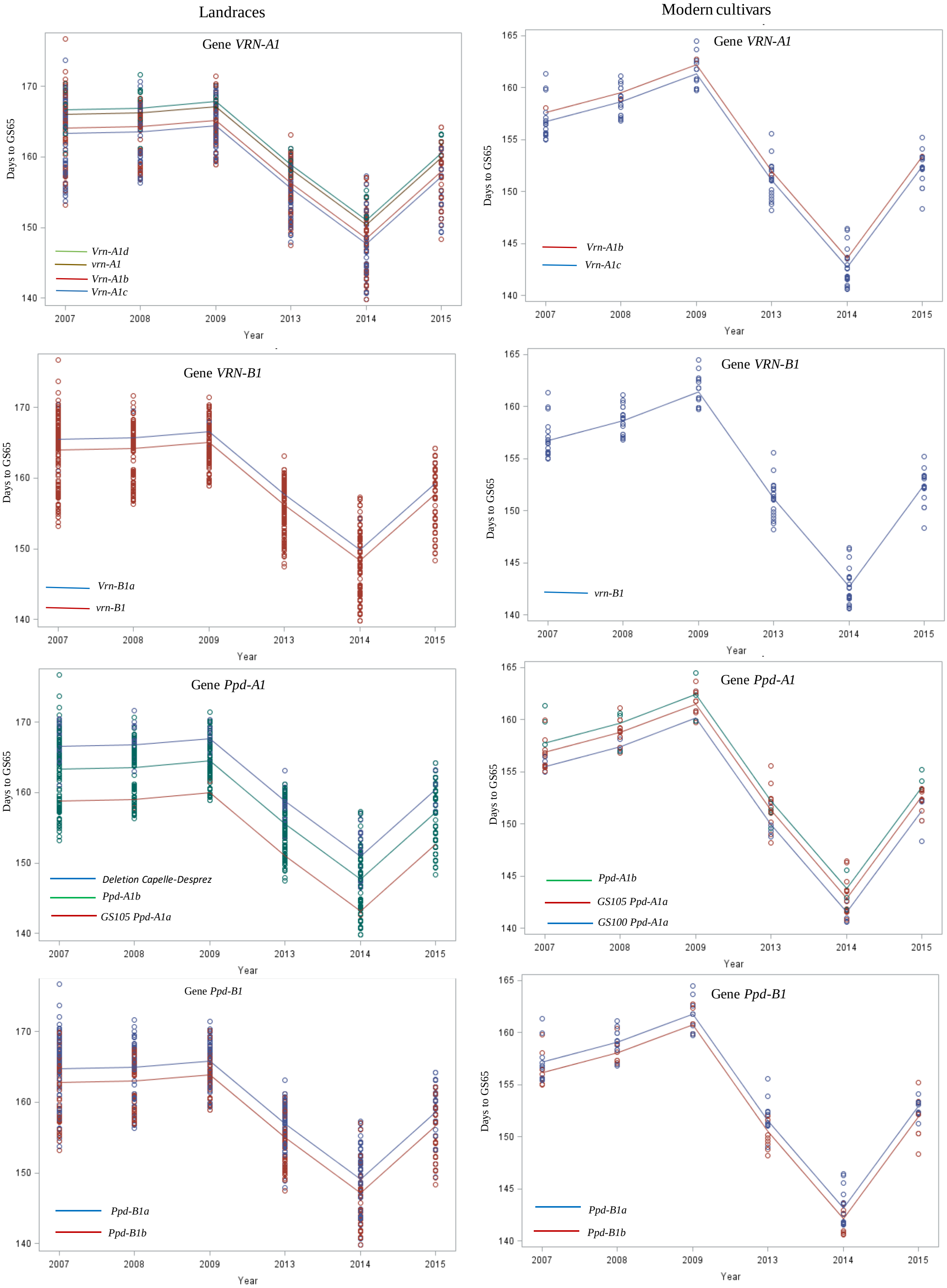

Supplement: FIGURE S2 — Individual alleles at each gene × year interaction for the number of days to GS65 (anthesis) in landraces (left) and modern cultivars (right). [file Image_2.pdf]
