# Supplementary material for: Allelic Variation at the Vernalization Response (Vrn-1) and Photoperiod Sensitivity (Ppd-1) Genes and Their Association With the Development of Durum Wheat Landraces and Modern Cultivars
Source: Front Plant Sci. 2020 Jun 23;11:838. doi: 10.3389/fpls.2020.00838 (PMC7325763; doi:10.3389/fpls.2020.00838)

SUPPLEMENTARY FIGURE S3. Allele combinations x year interaction for the number of days to GS65 (anthesis) in landraces (left) and modern cultivars (right). A) Allele combinations of *Vrn-1* genes; B) Allele combinations of *Ppd-1* genes; C) Allele combinations of *Vrn-1* + *Ppd-1* genes. See Table 6 for the meaning of allele combination (AC) numbers.

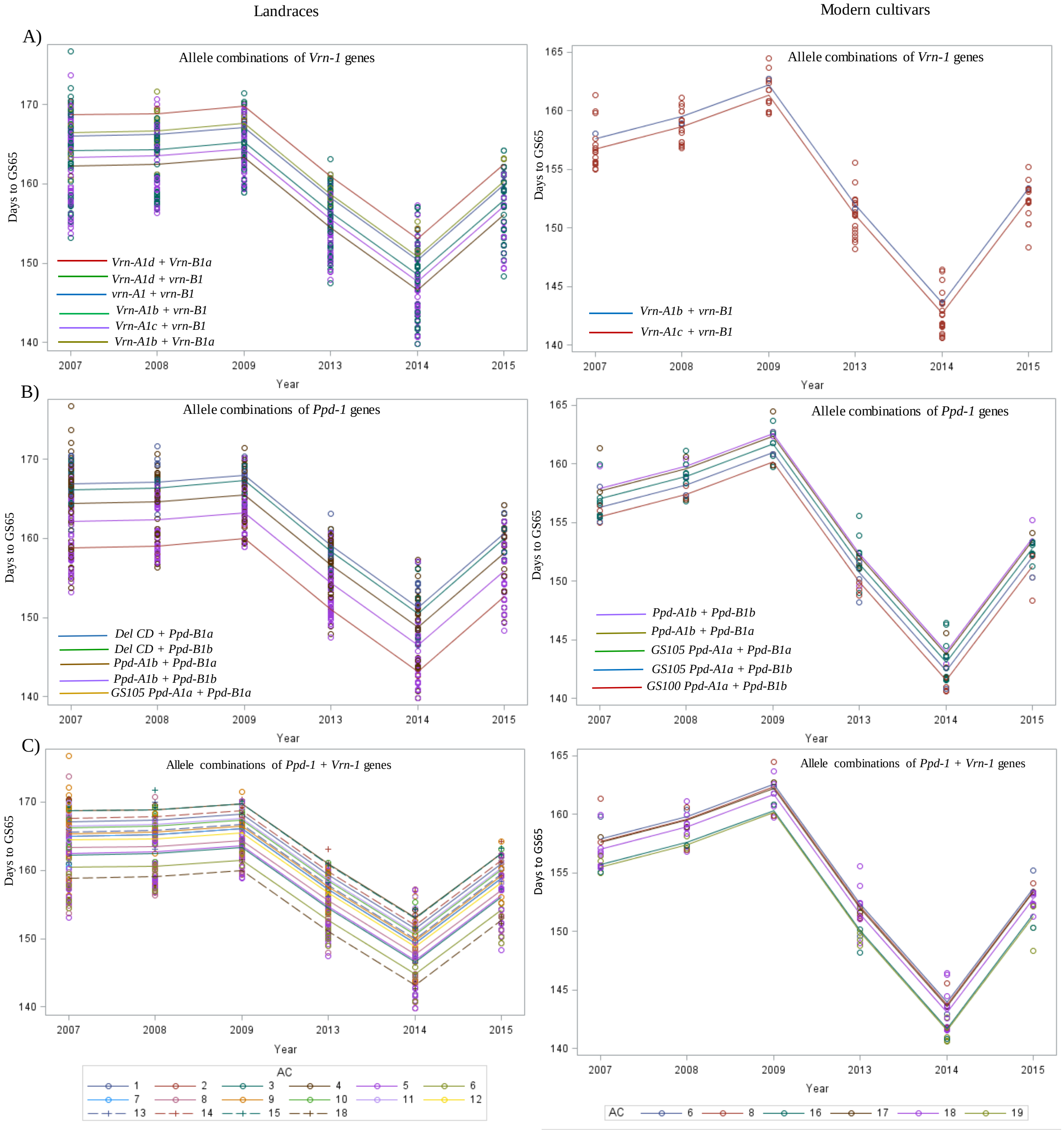

Supplement: FIGURE S3 — Allele combinations × year interaction for the number of days to GS65 (anthesis) in landraces (left) and modern cultivars (right). (A) Allele combinations of Vrn-1 genes; (B) allele combinations of Ppd-1 genes; (C) allele combinations of Vrn-1 + Ppd-1 genes. See Table 6 for the meaning of allele combination (AC) numbers. [file Image_3.pdf]
