# Supplementary material for: Allelic Variation at the Vernalization Response (Vrn-1) and Photoperiod Sensitivity (Ppd-1) Genes and Their Association With the Development of Durum Wheat Landraces and Modern Cultivars
Source: Front Plant Sci. 2020 Jun 23;11:838. doi: 10.3389/fpls.2020.00838 (PMC7325763; doi:10.3389/fpls.2020.00838)
Supplement: TABLE S1 — Allelic variants of vernalization and photoperiod sensitivity genes on each genotype of the durum wheat collection studied. [file Table_1.DOCX]

**SUPPLEMENTARY TABLE S1**. Allelic variants of vernalization and photoperiod sensitivity genes on each genotype of the durum wheat collection studied.

| **Type** | **Cultivar** | **Country/ Origin** | ***Vrn-A1*** | ***Vrn-B1*** | ***Ppd-A1*** | ***Ppd-B1*** |
| --- | --- | --- | --- | --- | --- | --- |
| Landrace | IG-92895 | Algeria | *Vrn-A1c* | *vrn-B1* | *DelCD* | *Ppd-B1b* |
| Landrace | IG-92967 | Algeria | *Vrn-A1c* | *vrn-B1* | *Ppd-A1b* | *Ppd-B1a* |
| Landrace | IG-93030 | Algeria | *Vrn-A1b* | *vrn-B1* | *Ppd-A1b* | *Ppd-B1b* |
| Landrace | IG-93621 | Algeria | *Vrn-A1b* | *vrn-B1* | *Ppd-A1b* | *Ppd-B1b* |
| Landrace | IG-94009 | Algeria | *Vrn-A1c* | *vrn-B1* | *DelCD* | *Ppd-B1b* |
| Landrace | Dur de Medeah | Algeria | *Vrn-A1c* | *vrn-B1* | *Ppd-A1b* | *Ppd-B1a* |
| Landrace | Tchirpan | Bulgaria | *Vrn-A1b* | *vrn-B1* | *Ppd-A1b* | *Ppd-B1a* |
| Landrace | Lozen 76 | Bulgaria | *Vrn-A1b* | *vrn-B1* | *Ppd-A1b* | *Ppd-B1a* |
| Landrace | Dalmatia 1 | Croatia | *Vrn-A1d* | *vrn-B1* | *Ppd-A1b* | *Ppd-B1a* |
| Landrace | Dalmatia 3 | Croatia | *Vrn-A1d* | *Vrn-B1a* | *DelCD* | *Ppd-B1a* |
| Landrace | 440-IX/96 | Croatia | *Vrn-A1d* | *vrn-B1* | *Ppd-A1b* | *Ppd-B1a* |
| Landrace | 441-IX/97 | Croatia | *Vrn-A1d* | *vrn-B1* | *Ppd-A1b* | *Ppd-B1a* |
| Landrace | Vroulos | Cyprus | *Vrn-A1b* | *vrn-B1* | *Ppd-A1b* | *Ppd-B1a* |
| Landrace | IG-82549 | Cyprus | *Vrn-A1b* | *vrn-B1* | *Ppd-A1b* | *Ppd-B1b* |
| Landrace | Muri | Cyprus | *Vrn-A1b* | *vrn-B1* | *Ppd-A1b* | *Ppd-B1b* |
| Landrace | FAO 29.912 | Cyprus | *Vrn-A1b* | *vrn-B1* | *Ppd-A1b* | *Ppd-B1b* |
| Landrace | Milagro | Egypt | *Vrn-A1d* | *vrn-B1* | *Ppd-A1b* | *Ppd-B1b* |
| Landrace | Reading | Egypt | *Vrn-A1b* | *vrn-B1* | *Ppd-A1b* | *Ppd-B1b* |
| Landrace | D-2 | Egypt | *Vrn-A1c* | *vrn-B1* | *Ppd-A1b* | *Ppd-B1a* |
| Landrace | 5P4 | Egypt | *Vrn-A1c* | *vrn-B1* | *DelCD* | *Ppd-B1a* |
| Landrace | 1P1 | Egypt | *Vrn-A1c* | *vrn-B1* | *GS105* | *Ppd-B1a* |
| Landrace | 2751 | Egypt | *Vrn-A1c* | *vrn-B1* | *Ppd-A1b* | *Ppd-B1a* |
| Landrace | MG 26429 | Egypt | *Vrn-A1c* | *vrn-B1* | *Ppd-A1b* | *Ppd-B1a* |
| Landrace | 31 | Egypt | *Vrn-A1c* | *vrn-B1* | *Ppd-A1b* | *Ppd-B1a* |
| Landrace | Sinai No.8 | Egypt | *Vrn-A1b* | *vrn-B1* | *Ppd-A1b* | *Ppd-B1a* |
| Landrace | Mishriki | Egypt | *Vrn-A1c* | *vrn-B1* | *Ppd-A1b* | *Ppd-B1b* |
| Landrace | Girgeh | Egypt | *Vrn-A1c* | *vrn-B1* | *Ppd-A1b* | *Ppd-B1a* |
| Landrace | Beladi Rouge | France | *Vrn-A1c* | *vrn-B1* | *DelCD* | *Ppd-B1a* |
| Landrace | De Santa Marta | France | *Vrn-A1c* | *vrn-B1* | *DelCD* | *Ppd-B1b* |
| Landrace | Iumillo | France | *Vrn-A1c* | *vrn-B1* | *Ppd-A1b* | *Ppd-B1a* |
| Landrace | Tounse | France | *Vrn-A1c* | *vrn-B1* | *DelCD* | *Ppd-B1b* |
| Landrace | Trigo Glutinoso | France | *Vrn-A1c* | *vrn-B1* | *Ppd-A1b* | *Ppd-B1a* |
| Landrace | Rubio enlargado d’Atlemteje | France | *Vrn-A1c* | *vrn-B1* | *DelCD* | *Ppd-B1b* |
| Landrace | IG-96802 | Greece | *Vrn-A1b* | *vrn-B1* | *Ppd-A1b* | *Ppd-B1a* |
| Landrace | IG-96851 | Greece | *Vrn-A1c* | *vrn-B1* | *Ppd-A1b* | *Ppd-B1a* |
| Landrace | Mavraani | Greece | *Vrn-A1c* | *vrn-B1* | *DelCD* | *Ppd-B1b* |
| Landrace | Rapsani | Greece | *Vrn-A1c* | *vrn-B1* | *Ppd-A1b* | *Ppd-B1b* |
| Landrace | Abu Fashit | Israel | *Vrn-A1c* | *vrn-B1* | *Ppd-A1b* | *Ppd-B1a* |
| Landrace | Etith | Israel | *Vrn-A1b* | *vrn-B1* | *Ppd-A1b* | *Ppd-B1b* |
| Landrace | Juljulith | Israel | *Vrn-A1c* | *vrn-B1* | *Ppd-A1b* | *Ppd-B1b* |
| Landrace | Hati | Israel | *Vrn-A1b* | *vrn-B1* | *Ppd-A1b* | *Ppd-B1b* |
| Landrace | JM-3987 | Israel | *Vrn-A1c* | *vrn-B1* | *Ppd-A1b* | *Ppd-B1a* |
| Landrace | JM-3989 | Israel | *Vrn-A1c* | *vrn-B1* | *Ppd-A1b* | *Ppd-B1b* |
| Landrace | Carlantino | Italy | *vrn-A1* | *vrn-B1* | *DelCD* | *Ppd-B1b* |
| Landrace | Cicirelo | Italy | *Vrn-A1b* | *vrn-B1* | *Ppd-A1b* | *Ppd-B1a* |
| Landrace | IG-83920 | Italy | *Vrn-A1b* | *vrn-B1* | *Ppd-A1b* | *Ppd-B1a* |
| Landrace | Carlo jucci | Italy | *Vrn-A1c* | *vrn-B1* | *GS105* | *Ppd-B1a* |
| Landrace | Senatore Capelli | Italy | *Vrn-A1b* | *vrn-B1* | *Ppd-A1b* | *Ppd-B1b* |
| Landrace | Hymera | Italy | *Vrn-A1c* | *vrn-B1* | *Ppd-A1b* | *Ppd-B1b* |
| Landrace | Trinakria | Italy | *Vrn-A1c* | *vrn-B1* | *Ppd-A1b* | *Ppd-B1a* |
| Landrace | Aziziah 17/45 | Italy | *Vrn-A1c* | *vrn-B1* | *Ppd-A1b* | *Ppd-B1b* |
| Landrace | Razza 208 | Italy | *Vrn-A1b* | *vrn-B1* | *Ppd-A1b* | *Ppd-B1b* |
| Landrace | Balilla Falso | Italy | *Vrn-A1c* | *vrn-B1* | *Ppd-A1b* | *Ppd-B1a* |
| Landrace | Milazzo | Italy | *Vrn-A1c* | *vrn-B1* | *Ppd-A1b* | *Ppd-B1a* |
| Landrace | Razza 181 | Italy | *Vrn-A1c* | *vrn-B1* | *Ppd-A1b* | *Ppd-B1a* |
| Landrace | Capeiti | Italy | *Vrn-A1c* | *vrn-B1* | *Ppd-A1b* | *Ppd-B1a* |
| Landrace | Safra Jerash | Jordan | *Vrn-A1b* | *vrn-B1* | *Ppd-A1b* | *Ppd-B1b* |
| Landrace | Harani Auttma | Jordan | *Vrn-A1b* | *vrn-B1* | *Ppd-A1b* | *Ppd-B1b* |
| Landrace | Salti na Zinia | Jordan | *Vrn-A1c* | *vrn-B1* | *Ppd-A1b* | *Ppd-B1b* |
| Landrace | Horani Howawi | Jordan | *Vrn-A1b* | *vrn-B1* | *Ppd-A1b* | *Ppd-B1b* |
| Landrace | Zugbieh Sutra | Jordan | *Vrn-A1c* | *vrn-B1* | *Ppd-A1b* | *Ppd-B1b* |
| Landrace | Zoghbiyeh Safra | Jordan | *Vrn-A1b* | *vrn-B1* | *Ppd-A1b* | *Ppd-B1b* |
| Landrace | 26 | Jordan | *Vrn-A1b* | *vrn-B1* | *Ppd-A1b* | *Ppd-B1b* |
| Landrace | IG-84856 | Lebanon | *Vrn-A1b* | *vrn-B1* | *Ppd-A1b* | *Ppd-B1a* |
| Landrace | 9923 | Lebanon | *Vrn-A1b* | *vrn-B1* | *Ppd-A1b* | *Ppd-B1b* |
| Landrace | 9929 | Lebanon | *Vrn-A1c* | *vrn-B1* | *Ppd-A1b* | *Ppd-B1b* |
| Landrace | 9935 | Lebanon | *Vrn-A1b* | *vrn-B1* | *Ppd-A1b* | *Ppd-B1a* |
| Landrace | 9918 | Lebanon | *Vrn-A1b* | *vrn-B1* | *Ppd-A1b* | *Ppd-B1b* |
| Landrace | Hourah | Lebanon | *Vrn-A1b* | *vrn-B1* | *Ppd-A1b* | *Ppd-B1b* |
| Landrace | Tripshiro | Libya | *Vrn-A1c* | *vrn-B1* | *Ppd-A1b* | *Ppd-B1b* |
| Landrace | 248-VII/7 | Macedonia | *Vrn-A1b* | *vrn-B1* | *DelCD* | *Ppd-B1a* |
| Landrace | 259-VII/12 | Macedonia | *Vrn-A1b* | *vrn-B1* | *Ppd-A1b* | *Ppd-B1a* |
| Landrace | VII/13-X11 | Macedonia | *Vrn-A1b* | *vrn-B1* | *Ppd-A1b* | *Ppd-B1a* |
| Landrace | 196/71 | Macedonia | *Vrn-A1b* | *vrn-B1* | *Ppd-A1b* | *Ppd-B1a* |
| Landrace | II/4 | Macedonia | *Vrn-A1b* | *vrn-B1* | *Ppd-A1b* | *Ppd-B1a* |
| Landrace | 356-I/9 | Montenegro | *Vrn-A1d* | *vrn-B1* | *Ppd-A1b* | *Ppd-B1a* |
| Landrace | 23 | Montenegro | *Vrn-A1d* | *vrn-B1* | *DelCD* | *Ppd-B1a* |
| Landrace | 33 | Montenegro | *Vrn-A1d* | *vrn-B1* | *DelCD* | *Ppd-B1a* |
| Landrace | 37 | Montenegro | *Vrn-A1b* | *vrn-B1* | *Ppd-A1b* | *Ppd-B1b* |
| Landrace | 42 | Montenegro | *Vrn-A1b* | *vrn-B1* | *Ppd-A1b* | *Ppd-B1a* |
| Landrace | Zoco Yebel Hebil | Morocco | *Vrn-A1d* | *vrn-B1* | *Ppd-A1b* | *Ppd-B1b* |
| Landrace | Maghoussa | Morocco | *Vrn-A1b* | *vrn-B1* | *Ppd-A1b* | *Ppd-B1b* |
| Landrace | Merzaga | Morocco | *Vrn-A1b* | *vrn-B1* | *Ppd-A1b* | *Ppd-B1b* |
| Landrace | Red Beard | Morocco | *Vrn-A1b* | *vrn-B1* | *Ppd-A1b* | *Ppd-B1b* |
| Landrace | Morocco | Morocco | *Vrn-A1c* | *vrn-B1* | *DelCD* | *Ppd-B1a* |
| Landrace | Saffi | Morocco | *Vrn-A1c* | *vrn-B1* | *DelCD* | *Ppd-B1a* |
| Landrace | Ble Dur 250 | Morocco | *Vrn-A1b* | *vrn-B1* | *Ppd-A1b* | *Ppd-B1b* |
| Landrace | Oned Zenati | Morocco | *Vrn-A1b* | *vrn-B1* | *Ppd-A1b* | *Ppd-B1b* |
| Landrace | Mahmoudi C | Morocco | *Vrn-A1b* | *vrn-B1* | *Ppd-A1b* | *Ppd-B1b* |
| Landrace | Maghoussa Amizmiz | Morocco | *Vrn-A1d* | *vrn-B1* | *Ppd-A1b* | *Ppd-B1a* |
| Landrace | Cobros | Morocco | *Vrn-A1b* | *vrn-B1* | *Ppd-A1b* | *Ppd-B1b* |
| Landrace | Marques | Portugal | *Vrn-A1c* | *vrn-B1* | *DelCD* | *Ppd-B1b* |
| Landrace | Raposinho | Portugal | *Vrn-A1c* | *vrn-B1* | *Ppd-A1b* | *Ppd-B1a* |
| Landrace | Durazio Rijo | Portugal | *Vrn-A1b* | *vrn-B1* | *Ppd-A1b* | *Ppd-B1a* |
| Landrace | Raspinegro | Portugal | *Vrn-A1c* | *vrn-B1* | *Ppd-A1b* | *Ppd-B1a* |
| Landrace | Anafil | Portugal | *Vrn-A1c* | *vrn-B1* | *DelCD* | *Ppd-B1a* |
| Landrace | Espanhol | Portugal | *Vrn-A1c* | *vrn-B1* | *DelCD* | *Ppd-B1a* |
| Landrace | Dezassete | Portugal | *Vrn-A1b* | *vrn-B1* | *DelCD* | *Ppd-B1a* |
| Landrace | Durazio Rijo Glabro | Portugal | *Vrn-A1c* | *vrn-B1* | *Ppd-A1b* | *Ppd-B1a* |
| Landrace | Amarelo Barba Preta | Portugal | *Vrn-A1c* | *vrn-B1* | *Ppd-A1b* | *Ppd-B1b* |
| Landrace | Alentejo | Portugal | *Vrn-A1b* | *vrn-B1* | *Ppd-A1b* | *Ppd-B1a* |
| Landrace | Caxudo de sete espigas | Portugal | *Vrn-A1b* | *vrn-B1* | *Ppd-A1b* | *Ppd-B1b* |
| Landrace | Tremes rijo | Portugal | *Vrn-A1b* | *vrn-B1* | *DelCD* | *Ppd-B1b* |
| Landrace | Lobeiro de grao escuro | Portugal | *Vrn-A1b* | *vrn-B1* | *DelCD* | *Ppd-B1a* |
| Landrace | Belgrade 9 | Serbia | *Vrn-A1b* | *vrn-B1* | *DelCD* | *Ppd-B1a* |
| Landrace | 1575 | Serbia | *Vrn-A1b* | *vrn-B1* | *DelCD* | *Ppd-B1a* |
| Landrace | 18/71 | Serbia | *Vrn-A1c* | *vrn-B1* | *Ppd-A1b* | *Ppd-B1b* |
| Landrace | Arisnegro de Tenerife | Spain | *Vrn-A1c* | *vrn-B1* | *Ppd-A1b* | *Ppd-B1b* |
| Landrace | Basto Duro | Spain | *Vrn-A1c* | *vrn-B1* | *Ppd-A1b* | *Ppd-B1a* |
| Landrace | Blanquillo | Spain | *Vrn-A1b* | *vrn-B1* | *Ppd-A1b* | *Ppd-B1a* |
| Landrace | Candeal de Salamanca | Spain | *Vrn-A1c* | *vrn-B1* | *Ppd-A1b* | *Ppd-B1a* |
| Landrace | Colorado de Jerez | Spain | *Vrn-A1c* | *vrn-B1* | *Ppd-A1b* | *Ppd-B1a* |
| Landrace | Enano de Andújar | Spain | *Vrn-A1b* | *vrn-B1* | *Ppd-A1b* | *Ppd-B1b* |
| Landrace | Fartó | Spain | *Vrn-A1c* | *vrn-B1* | *Ppd-A1b* | *Ppd-B1a* |
| Landrace | Gros de Cerdaña | Spain | *Vrn-A1b* | *vrn-B1* | *DelCD* | *Ppd-B1a* |
| Landrace | Heraldo del Rhin | Spain | *Vrn-A1c* | *vrn-B1* | *DelCD* | *Ppd-B1a* |
| Landrace | Pinet | Spain | *Vrn-A1c* | *vrn-B1* | *Ppd-A1b* | *Ppd-B1a* |
| Landrace | Pisana cañihueca | Spain | *Vrn-A1b* | *vrn-B1* | *DelCD* | *Ppd-B1a* |
| Landrace | Raspinegro Canario | Spain | *Vrn-A1c* | *vrn-B1* | *Ppd-A1b* | *Ppd-B1a* |
| Landrace | Raspinegro de Alcalá Guadaira | Spain | *Vrn-A1c* | *vrn-B1* | *DelCD* | *Ppd-B1a* |
| Landrace | Recio de Almería | Spain | *Vrn-A1b* | *vrn-B1* | *Ppd-A1b* | *Ppd-B1a* |
| Landrace | Verdial | Spain | *vrn-A1* | *vrn-B1* | *Ppd-A1b* | *Ppd-B1b* |
| Landrace | Andalucía 344 | Spain | *Vrn-A1c* | *vrn-B1* | *Ppd-A1b* | *Ppd-B1a* |
| Landrace | Azulejo de Villa del Río | Spain | *Vrn-A1b* | *Vrn-B1a* | *Ppd-A1b* | *Ppd-B1a* |
| Landrace | Blancal | Spain | *Vrn-A1b* | *vrn-B1* | *Ppd-A1b* | *Ppd-B1a* |
| Landrace | Blanquillón de Boñar | Spain | *Vrn-A1c* | *vrn-B1* | *DelCD* | *Ppd-B1b* |
| Landrace | Claro de Balazote | Spain | *Vrn-A1c* | *vrn-B1* | *DelCD* | *Ppd-B1b* |
| Landrace | Entrelargo de Montijo | Spain | *Vrn-A1c* | *vrn-B1* | *Ppd-A1b* | *Ppd-B1a* |
| Landrace | Farto cañifino | Spain | *Vrn-A1c* | *vrn-B1* | *Ppd-A1b* | *Ppd-B1a* |
| Landrace | Rubio de Montijo | Spain | *Vrn-A1c* | *vrn-B1* | *Ppd-A1b* | *Ppd-B1a* |
| Landrace | Semental | Spain | *Vrn-A1c* | *vrn-B1* | *Ppd-A1b* | *Ppd-B1a* |
| Landrace | Recio de Cañete | Spain | *Vrn-A1b* | *vrn-B1* | *Ppd-A1b* | *Ppd-B1a* |
| Landrace | IG-95812 | Syria | *Vrn-A1b* | *vrn-B1* | *Ppd-A1b* | *Ppd-B1b* |
| Landrace | IG-95841 | Syria | *Vrn-A1c* | *vrn-B1* | *DelCD* | *Ppd-B1a* |
| Landrace | IG-95847 | Syria | *Vrn-A1b* | *vrn-B1* | *Ppd-A1b* | *Ppd-B1b* |
| Landrace | IG-95931 | Syria | *Vrn-A1c* | *vrn-B1* | *Ppd-A1b* | *Ppd-B1a* |
| Landrace | Louri AP 5 | Tunisia | *Vrn-A1b* | *vrn-B1* | *Ppd-A1b* | *Ppd-B1a* |
| Landrace | Souri | Tunisia | *Vrn-A1c* | *vrn-B1* | *Ppd-A1b* | *Ppd-B1b* |
| Landrace | Realforte | Tunisia | *Vrn-A1b* | *vrn-B1* | *Ppd-A1b* | *Ppd-B1b* |
| Landrace | Hamira | Tunisia | *Vrn-A1b* | *vrn-B1* | *Ppd-A1b* | *Ppd-B1b* |
| Landrace | Mindium | Turkey | *Vrn-A1b* | *vrn-B1* | *Ppd-A1b* | *Ppd-B1b* |
| Landrace | BGE-018192 | Turkey | *Vrn-A1b* | *vrn-B1* | *Ppd-A1b* | *Ppd-B1a* |
| Landrace | BGE018351 | Turkey | *Vrn-A1b* | *vrn-B1* | *Ppd-A1b* | *Ppd-B1a* |
| Landrace | BGE018353 | Turkey | *Vrn-A1b* | *vrn-B1* | *Ppd-A1b* | *Ppd-B1a* |
| Landrace | BGE-018354 | Turkey | *Vrn-A1b* | *vrn-B1* | *Ppd-A1b* | *Ppd-B1a* |
| Landrace | BGE019262 | Turkey | *Vrn-A1b* | *vrn-B1* | *Ppd-A1b* | *Ppd-B1a* |
| Landrace | BGE-019263 | Turkey | *Vrn-A1c* | *vrn-B1* | *Ppd-A1b* | *Ppd-B1a* |
| Landrace | BGE019264 | Turkey | *Vrn-A1b* | *vrn-B1* | *Ppd-A1b* | *Ppd-B1a* |
| Landrace | BGE019265 | Turkey | *Vrn-A1b* | *vrn-B1* | *Ppd-A1b* | *Ppd-B1b* |
| Landrace | BGE019266 | Turkey | *Vrn-A1b* | *vrn-B1* | *Ppd-A1b* | *Ppd-B1a* |
| Landrace | BGE-019270 | Turkey | *Vrn-A1b* | *vrn-B1* | *Ppd-A1b* | *Ppd-B1b* |
| Modern | Gallareta | CIMMYT | *Vrn-A1c* | *vrn-B1* | *GS105* | *Ppd-B1a* |
| Modern | Jupare | CIMMYT | *Vrn-A1c* | *vrn-B1* | *GS105* | *Ppd-B1a* |
| Modern | Sula | CIMMYT | *Vrn-A1c* | *vrn-B1* | *GS105* | *Ppd-B1a* |
| Modern | Vitron | CIMMYT | *Vrn-A1c* | *vrn-B1* | *GS105* | *Ppd-B1a* |
| Modern | Arment | France | *Vrn-A1c* | *vrn-B1* | *Ppd-A1b* | *Ppd-B1b* |
| Modern | Claudio | Italy | *Vrn-A1b* | *vrn-B1* | *GS105* | *Ppd-B1b* |
| Modern | Meridiano | Italy | *Vrn-A1c* | *vrn-B1* | *GS105* | *Ppd-B1a* |
| Modern | Simeto | Italy | *Vrn-A1c* | *vrn-B1* | *Ppd-A1b* | *Ppd-B1a* |
| Modern | Svevo | Italy | *Vrn-A1c* | *vrn-B1* | *GS105* | *Ppd-B1b* |
| Modern | Amilcar | Spain | *Vrn-A1c* | *vrn-B1* | *GS105* | *Ppd-B1b* |
| Modern | Ancalei | Spain | *Vrn-A1c* | *vrn-B1* | *GS105* | *Ppd-B1a* |
| Modern | Astigi | Spain | *Vrn-A1c* | *vrn-B1* | *GS105* | *Ppd-B1a* |
| Modern | Boabdil | Spain | *Vrn-A1c* | *vrn-B1* | *GS105* | *Ppd-B1a* |
| Modern | Bolido | Spain | *Vrn-A1c* | *vrn-B1* | *GS100* | *Ppd-B1b* |
| Modern | Bolo | Spain | *Vrn-A1c* | *vrn-B1* | *Ppd-A1b* | *Ppd-B1a* |
| Modern | Hispasano | Spain | *Vrn-A1c* | *vrn-B1* | *GS105* | *Ppd-B1a* |
| Modern | Senadur | Spain | *Vrn-A1c* | *vrn-B1* | *GS100* | *Ppd-B1b* |
| Modern | Vitronero | Spain | *Vrn-A1c* | *vrn-B1* | *GS105* | *Ppd-B1a* |
| Modern | Kronos | USA | *Vrn-A1c* | *vrn-B1* | *GS100* | *Ppd-B1b* |
| Modern | Ocotillo | USA | *Vrn-A1c* | *vrn-B1* | *GS100* | *Ppd-B1b* |
