# Supplementary material for: Allelic Variation at the Vernalization Response (Vrn-1) and Photoperiod Sensitivity (Ppd-1) Genes and Their Association With the Development of Durum Wheat Landraces and Modern Cultivars
Source: Front Plant Sci. 2020 Jun 23;11:838. doi: 10.3389/fpls.2020.00838 (PMC7325763; doi:10.3389/fpls.2020.00838)
Supplement: TABLE S2 — Allelic variants of vernalization and photoperiod sensitivity genes observed in durum wheat. [file Table_2.DOCX]

**SUPPLEMENTARY TABLE S2**. Allelic variants of vernalization and photoperiod sensitivity genes observed in durum wheat.

^1)^ Nomenclature described in Wilhelm *et al*. (2009)
