# Supplementary material for: Allelic Variation at the Vernalization Response (Vrn-1) and Photoperiod Sensitivity (Ppd-1) Genes and Their Association With the Development of Durum Wheat Landraces and Modern Cultivars
Source: Front Plant Sci. 2020 Jun 23;11:838. doi: 10.3389/fpls.2020.00838 (PMC7325763; doi:10.3389/fpls.2020.00838)
Supplement: TABLE S3 — Marker summary. [file Table_3.DOCX]

**SUPPLEMENTARY TABLE S3**. Marker summary.
